# Signal mining and safety profile analysis of lapatinib: a pharmacovigilance analysis of the FDA Adverse Event Reporting System (FAERS) database

**DOI:** 10.1080/20523211.2025.2611182

**Published:** 2026-01-13

**Authors:** Emelith Cerbito, Mohammad Issam Diab, Ali Alhoshani, Zaid H. Maayah

**Affiliations:** a Department of Pharmaceutical Sciences, College of Pharmacy, QU Health, Qatar University, Doha, Qatar; b Clinical Pharmacy and Practice Department, College of Pharmacy, QU Health, Qatar University, Doha, Qatar; c Department of Pharmacology & Toxicology, College of Pharmacy, King Saud University, Riyadh, Saudi Arabia

**Keywords:** Lapatinib, FAERS database, adverse events, drug safety, pharmacovigilance, drug-induced toxicity, disproportionality analysis

## Abstract

**Background:**

There remains a gap in understanding lapatinib's real-world safety, particularly in rare adverse events (AEs). Thus, this study aims to evaluate lapatinib’s safety by (1) performing data mining of the FDA Adverse Event Reporting System (FAERS); and (2) detecting and analysing safety signals associated with lapatinib that may require monitoring.

**Methods:**

FAERS data from March 2007 to July 2024 were analysed via OpenVigil (version 2.1). AEs were categorised into preferred terms (PTs) and system organ classes (SOCs) using the Medical Dictionary for Regulatory Activities. We used descriptive analysis to analyse report characteristics and four signal detection algorithms to quantify risk signals, including Proportional Reporting Ratio (PRR), Reporting Odds Ratio (ROR), Multi-item Gamma Poisson Shrinker (MGPS), and Bayesian Confidence Propagation Neural Network (BCPNN). Top novel strong suspected AEs were further assessed using a case-by-case analysis. The Naranjo algorithm was utilised to determine the potential relation between the suspected AEs and lapatinib.

**Results:**

From 25,506,744 retrieved reports, 18,407 PTs identified lapatinib as the primary suspect, resulting in 10,959 signals analysed. AEs were predominantly females (77.9%) and individuals aged 18–64 (45.38%). Lapatinib-induced AEs affected 16 systems, with 155 lapatinib-related PTs; 115 of these were significantly disproportionate, including 57 new PTs. While gastrointestinal and dermatological disorders were the most common, the latter was more strongly associated with lapatinib, with diarrhoea being the only strong gastrointestinal signal. Notably, cardiac events were less reported, and the top new AEs based on signal strength, such as hypocapnia, lip ulceration, and hepatic infection, were mostly found to be ‘possibly’ related to lapatinib based on the case-by-case evaluation, warranting further clinical assessments. Initial or prolonged hospitalisation, death, and life-threatening events were the most common AEs outcomes reported, accounting for 28.79%, 13.79%, and 3.06%, respectively.

**Conclusion:**

This study provides valuable insights into lapatinib-induced toxicity in real-world settings.

## Background

Breast cancer (BC) is a life-threatening condition and stands as one of the most commonly diagnosed solid organ cancers in females worldwide (Afaya et al., 2023; Arnold et al., 2022; Courtney et al., 2022; Cox et al., 2006; Heer et al., 2020; Youlden et al., 2012). However, despite recent advancements in cancer therapy, several factors, such as high recurrence rates, poor prognosis, and expensive chemotherapy, have led to a significant rise in global rates of cancer-related mortality, making BC a public concern (Afaya et al., 2023; Courtney et al., 2022). According to the World Health Organization (WHO), nearly two million women worldwide were diagnosed with breast cancer in 2022, resulting in approximately 670,000 deaths (WHO, 2024). Furthermore, one of the key contributing factors to rapid tumour progression is the overexpression of human epidermal growth factor receptor type-2 (HER-2/ ErbB2) (Nader-Marta et al., 2022). It has been found that nearly 15% to 20% of BC cases overexpress HER-2 (Nader-Marta et al., 2022). As a result, several highly effective targeted chemotherapies have been developed, including lapatinib, to potentially combat this issue. Lapatinib is an oral, small molecule, dual tyrosine kinase inhibitor (TKI) that uniquely targets both the HER-2/ErbB2 and the epidermal growth factor receptor (HER-1/EGFR/ErbB1) (Oakman et al., 2010), making it a promising treatment option for trastuzumab-resistant patients with metastatic BC.

However, the use of lapatinib has been limited due to several safety concerns. Clinical trials have shown that it can cause a range of toxicities, including gastrointestinal (GI) issues (Baselga et al., 2012; Blackwell et al., 2010; Yang et al., 2018), skin reactions (Blackwell et al., 2010; Burris et al., 2005), and cardiac problems (Di Leo et al., 2008; Geyer et al., 2006). Adding to these findings, periodic safety surveillance conducted by pharmaceutical industries and market regulators, such as the US Food and Drug Administration (FDA) and the European Medicines Agency (EMA), has played a major role in identifying additional potential drug-related safety risks post-marketing, such as interstitial lung disease and pneumonitis (*Assessment report: Tyverb (EMA/515162/2018)*, 2018; Yamamoto et al., 2016). Recent pharmacovigilance studies on HER-2 inhibitors have also confirmed similar serious adverse events (AEs) associated with lapatinib (Bao et al., 2024; Barbieri et al., 2022; Waliany et al., 2023; Wittayanukorn et al., 2017; Xie et al., 2020), however, there still remains a lack of real-world data from academic peer-reviewed studies focusing on less commonly reported AEs associated with lapatinib alone that may be serious or life-threatening. Therefore, to address this, this research analyses safety reports from the FDA Adverse Event Reporting System (FAERS) database, aiming to provide additional insights into lapatinib's real-world safety that can potentially complement ongoing safety reviews by the FDA and pharmaceutical industries.

The FAERS database is a recognised spontaneous reporting system for AEs and provides valuable real-world data for monitoring AEs reported globally (Mo et al., 2023). Given the ongoing widespread use of lapatinib as an alternative agent for advanced BC and the importance of identifying AEs, this study, therefore, aims to evaluate the safety of lapatinib by (1) performing data mining of FAERS, and (2) detecting and analysing safety signals associated with lapatinib that may require adequate monitoring.

## Methods

### Data sources

Retrospective safety data on lapatinib were retrieved from the FAERS database from March 2007 to July 2024 using OpenVigil (version 2.1) online software (https://openvigil.sourceforge.net/) (Böhm et al., 2016). Spontaneous reporting systems for AEs, such as the FAERS database, serve as a crucial source of information for detecting AEs not identified in clinical trials (Noguchi et al., 2021; Sakaeda et al., 2013). These databases play a crucial role in monitoring and evaluating the safety of drug use in real-world practice (Emanuel et al., 2018). The FAERS database is a publicly accessible database of AEs safety reports that allows access to a diverse set of data on AEs reported around the world by various reporters, like healthcare practitioners (HCP) (like physicians, nurses, pharmacists, and others in allied health), pharmaceutical companies, and consumers (Meng et al., 2019). The FAERS dataset comprises 7 types of data, including (1) patient demographics, (2) drug information, (3) indications for use, (4) adverse drug reactions data, (5) patient outcomes data, (6) drug therapies information, and (7) report sources information (Yu et al., 2020). All safety reports submitted to FAERS are assessed via quantitative signal detection algorithms, in which any signal identified indicates a potential safety concern (Sakaeda et al., 2011). In addition, the FAERS database codes all AEs via terminologies from the Medical Dictionary for Regulatory Activities (MedDRA). OpenVigil, on the other hand, is an innovative pharmacovigilance analysis tool that can be accessed online and utilises the openFDA online interface to access pharmacovigilance data from the FAERS database (Böhm et al., 2016).

### Data mining

This study utilised OpenVigil to analyse entire cases truly related to lapatinib, in which a case contributed to the results if any of its reports included a lapatinib-event association. Moreover, we only selected adverse event reports from FAERS with the target drug, lapatinib, defined as the ‘Primary suspect’ using ‘ROLE_CODE’ assigned by reporters. Retrieved AEs data were then categorised and standardised using preferred terms (PTs) illustrated by the MedDRA (version 24.0) and were classified into various systems using the system organ classes (SOCs). Any reported adverse event not classified as a PT was excluded from the analysis. To ensure cleaned FDA data, duplicate AE reports were excluded according to the FDA's recommendation using OpenVigil's duplicate removal function prior to statistical analysis (Böhm et al., 2016). Patient outcomes for all lapatinib AEs were also extracted, which can be classified into 6 groups: (1) death, (2) disability, (3) initial or prolonged hospitalisation, (4) life-threatening, (5) required intervention, and (6) others.

Furthermore, based on the findings of the disproportionality analysis, a case-by-case assessment of FAERS reports was also conducted using the Naranjo adverse drug reaction probability scale (Naranjo et al., 1981) to further investigate patient characteristics and determine potential causal associations between lapatinib and new suspected AEs of moderate and/or strong signal intensity. OpenVigil provides the following data: Case ID, patient age, sex, reporting year, reporter country, concomitant drugs, dose administered, outcomes, and other adverse events. Each safety report was independently assessed by two raters (E.C. and Z.M.) Any discrepancies were resolved through discussion until consensus was reached. In case of unresolved conflicts, other co-authors (A.A and M.D.) were consulted. Each adverse event could be categorised as high probable or definite (score ≥ 9), probable (scores 5–8), possible (scores 1–4), or doubtful (score ≤ 0) (Naranjo et al., 1981).

### Statistical analysis

Descriptive analysis was employed to summarise the characteristics of retrieved AEs reports on lapatinib, while safety signal detection in patients treated with lapatinib was assessed using disproportionality analysis, which examines variations in the proportion of AE reports (Noguchi et al., 2021). Disproportionality analysis is widely employed in post-marketing surveillance databases to investigate potential links between drugs and AEs (Zhang et al., 2023). Some of the most commonly utilised signal detection algorithms in pharmacovigilance studies include Proportional Reporting Ratio (PRR), Reporting Odds Ratio (ROR), Multi-item Gamma Poisson Shrinker (MGPS), and Bayesian Confidence Propagation Neural Network (BCPNN) (Noguchi et al., 2021; Park et al., 2020). PRR and ROR are frequentist methods, while MGPS and BCPNN (the new information component (IC) method), are Bayesian methods (Park et al., 2020). Studies show that Bayesian methods are better than frequentist methods like PRR (Evans et al., 2001; Hu et al., 2015) as they provide a more stable estimation of drug-event associations, especially with a small number of reports (Roux et al., 2005; van de Schoot et al., 2014). Additionally, PRR and ROR are known to have a relatively higher sensitivity making it susceptible to false positive signals, unlike MGPS and BCPNN (Park et al., 2020; Zou et al., 2023). Nevertheless, ROR remains useful for minimising bias in instances where the number of reported events is limited (Rothman et al., 2004), whereas PRR is beneficial for identifying and assessing the influence of different risk factors, enabling a more effective risk detection (Evans et al., 2001). Thus, given the pros and cons of each algorithm, this study combined all four signal detection methods (PRR, ROR, MGPS, and BCPNN) to comprehensively detect signals of lapatinib and reduce the risk of overestimating or underestimating associations due to data bias. Additionally, 95% confidence intervals (CIs) were used to analyse reported AEs related to lapatinib. A reported signal was deemed a suspected AE statistically associated with lapatinib only if all four methods indicated positive results.

The equations used to calculate all algorithms, as well as their judgment criteria for statistical significance and signal intensity, are provided in Table 1 and Table 2. The criteria for positive safety signal detection are as follows: a) ROR, the lower limit of 95% CI must exceed 1, and the number of reports must be at least 3 (Cutroneo et al., 2024; Noguchi et al., 2019, 2021); (b) PRR, the lower limit of 95% CI must exceed 1, PRR ≥2, Chi-square (χ2) must be at least 4, and the number of reports must be at least 3 (Cutroneo et al., 2024; Evans et al., 2001; Noguchi et al., 2019); (c) BCPNN, the information component (IC), a measure of disproportionality that compares the observed and the expected reporting of a target drug-AE combination, must be positive, and its lower limit of 95% CI (IC025) must also be positive (Cutroneo et al., 2024; Noguchi et al., 2019, 2021); (d) MGPS, the Empirical Bayes Geometric Mean (EBGM) value must exceed 2 (Cutroneo et al., 2024; Noguchi et al., 2019, 2021). R software (version 4.4.2) and RStudio were used to conduct disproportionality analysis using the four algorithms, including IC025 of the BCPNN method to depict association. All data collected were inputted and analysed in Microsoft Excel (Version 16.92).

**Table 1.** Disproportionality analysis 2 × 2 contingency table (a number of reports with suspect adverse event of the suspect drug, b number of reports with all other adverse events of the suspect drug, c number of reports with the suspect adverse event of all other drugs, d number of reports with all other adverse events of all other drugs).

Item	Adverse events of interest	Other Adverse Events	Total
Drug of interest	a	b	a + b
Other drugs	c	d	c + d
Total	a + c	b + d	a + c + b + d

**Table 2.** Summary of algorithms utilised in risk signal detection (ROR reporting odds ratio, PRR proportional reporting ratio, BCPNN Bayesian confidence propagation neural network, MGPS multi-item gamma Poisson shrinker, IC Information component, IC025 lower limit of 95% CI of the IC, EBGM empirical Bayesian geometric mean, EBGM05 lower limit of 95% CI of EBGM) (Liu et al., 2025; Pan et al., 2024; Zou et al., 2023).

Algorithms	Equation	Judgement criteria for significance	Judgement criteria for signal intensity	Signal intensity
ROR	ROR = ad/bc 95%CI = e^ln(ROR)^ ^±^ ^1.96(1/a^ ^+^ ^1/b^ ^+^ ^1/c^ ^+^ ^1/d)^0.5^	Lower limit of 95% CI > 1, N ≥ 3	2 < ROR ≤ 10	Weak (+)
			10 < ROR ≤ 50	Medium (++)
			ROR > 50	Strong (+++)
PRR	PRR = (a(c + d))/(c(a + b)) χ^2^ = [(ad − bc)^2^](a + b + c + d)/[(a + b)(c + d)(a + c)(b + d)]	Lower limit of 95% CI > 1, PRR≥2, χ2 ≥ 4, N ≥ 3	2 < PRR ≤ 10	Weak (+)
			10 < PRR ≤ 50	Medium (++)
			PRR > 50	Strong (+++)
BCPNN	IC = log_2_a(a + b + c + d)/((a + c)(a + b)) IC_025_ = e^ln(IC)−1.96(1/a^ ^+^ ^1/b^ ^+^ ^1/c^ ^+^ ^1/d)^0.5^	IC > 0, IC_025_ > 0, N ≥ 3	0 < IC_025_ ≤ 1.5	Weak (+)
			1.5 < IC_025_ ≤ 3	Medium (++)
			IC_025_ > 3	Strong (+++)
MGPS	EBGM = a(a + b + c + d)/ ((a + c)/(a + b)) EBGM_05_ = e^ln(EBGM)−1.64(1/a^ ^+^ ^1/b^ ^+^ ^1/c^ ^+^ ^1/d)^0.5^	EBGM_05_ > 2, N > 0	2 < EBGM_05_ ≤ 10	Weak (+)
			10 < EBGM_05_ ≤ 30	Medium (++)
			EBGM_05_ > 30	Strong (+++)

## Results

### Descriptive analysis

**Figure 1.**
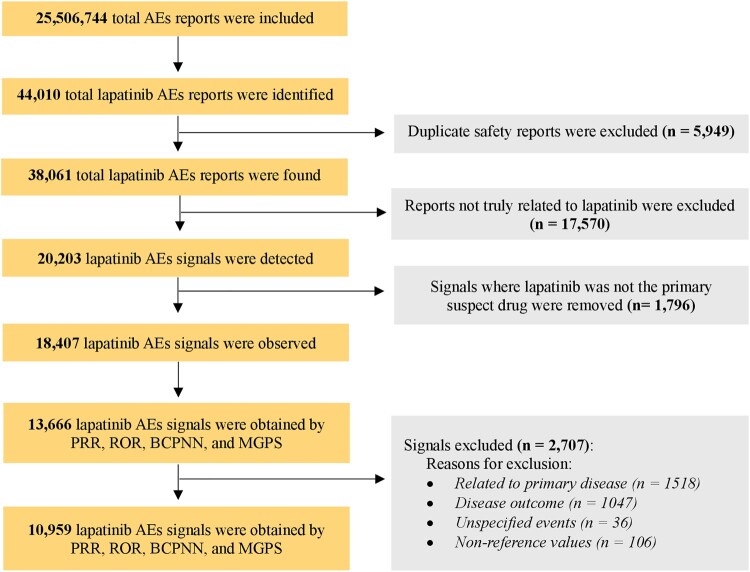
Flow diagram for adverse events identification from FAERS (AEs adverse events, PRR proportional reporting ratio, ROR reporting odds ratio, BCPNN Bayesian confidence propagation neural network, MGPS multi-item gamma poison shrinker).

From March 2007 to July 2024, 25,506,744 reports were submitted to FAERS. Among these, 44,010 were lapatinib-related (Figure 1). After eliminating duplicate reports, 38,061 unique AEs remained, involving 18,407 PTs where lapatinib was the primary suspect. Following an assessment of AE signals via ROR, PRR, BCPNN, and MGPS, a total of 13,666 potential risk signals were detected. However, 2,707 were deemed invalid, including those related to (1) primary diseases (e.g. disease progression); (2) disease outcomes (e.g. death); (3) non-reference values (e.g. investigation); and (4) unspecified events (e.g. oncologic complication). Ultimately, 10,959 positive signals were included in the study analysis (Figure 1).

The basic clinical characteristics of patients treated with lapatinib are summarised in Table 3. The present study revealed a predominance of female patients (77.90%, n = 29,650) compared to males (3.43%, n = 1304). The majority were adults aged 18 - 64 (45.38%, n = 17,273), while 16 cases involved patients less than 18 years old. Given lapatinib’s approved indication, it is not surprising to note that half of the cases reported BC as the primary indication for lapatinib (50.04%, n = 19,043). Among patients under 18 years of age, no clear therapeutic indication for lapatinib use was reported; however, two cases reported its use for adrenal gland cancer. Nevertheless, overall, in 23.39% of all reports, the indication for lapatinib treatment was not specified. Furthermore, many of the retrieved reports originated from North America (52.13%, n = 19,842), mainly from the United States (US), which accounted for 45.56% (n = 17,340) of the submitted AEs on lapatinib. This was followed by reports from Europe (19.38%, n = 7,377) and Asia (9.17%, n = 3,492). Regarding the number of reports over the past five years, the highest yields were recorded in 2019 and 2023 (Table 3).

**Table 3.** Clinical characteristics of adverse event reports from FAERS (March 2007 to July 2024).

Characteristic	Lapatinib, N (%)
**Total number of reports (N)**	38061
*Gender*	
Female	29650 (77.90)
Male	1304 (3.43)
Not specified	7107 (18.67)
*Age (years)*	
< 18	16 (0.04)
18–64	17273 (45.38)
≥ 65	4324 (11.36)
Not specified	16448 (43.21)
*Reporter regions*	
North America	19842 (52.13)
United states	17340 (45.56)
Europe	7377 (19.38)
Asia	3492 (9.17)
South America	852 (2.24)
Oceania	599 (1.57)
Africa	182 (0.48)
Not specified	5717 (15.02)
*Top Indications*	
Breast cancer	19043 (50.04%)
Breast cancer	12705 (33.38)
Breast cancer metastatic	5175 (13.60)
Breast cancer female	1163 (3.06)
Product used for unknown indication	6588 (17.31)
Drug use for unknown indication	2314 (6.08)
*Reporting year (5 years)*	
2023	1111 (2.92)
2022	660 (1.73)
2021	1015 (2.67)
2020	1020 (2.68)
2019	1396 (3.67)
*Outcomes*	
Death	5250 (13.79)
Disability	532 (1.40)
Hospitalisation (Initial or prolonged)	10958 (28.79)
Life-threatening	1164 (3.06)
Required intervention	41 (0.11)
Other serious outcomes	13968 (36.70)

Interestingly, all six outcomes associated with lapatinib-induced AEs were reported, with ‘Initial or prolonged hospitalization’ being the most frequently observed outcome, accounting for 28.79% (n = 10,958) of reported AEs. In contrast, ‘death’ and ‘life-threatening’ events accounted for 13.79% (n = 5,250) and 3.06% (n = 1,164), respectively (Table 3). Notably, the increasing rates of hospitalisation and death may be more attributable to tumour progression rather than solely to the adverse events related to lapatinib.

### Adverse event signals of system organ class

Using the MedDRA, all 10,959 detected signals were categorised based on the affected systems and organs. The signal strength of AEs of lapatinib at the system organ class (SOC) level is provided in Table 4. Overall, 16 organ systems were significantly involved in lapatinib-induced AEs, as identified by at least one of the four algorithms, including but not limited to blood and lymphatic system disorders (SOC:10005329), cardiac disorders (SOC:10007541), gastrointestinal disorders (SOC: 10017947), general disorders and administration site conditions (SOC:10018065), hepatobiliary disorders (SOC:10019805), infections and infestations (SOC:10021881), injury, poisoning and procedural complications (SOC:10022117), metabolism and nutrition disorders (SOC:10027433), skin and subcutaneous tissue disorders (SOC:10040785), and respiratory, thoracic and mediastinal disorders (SOC: 10038738).

**Table 4.** Total number of cases reported according to SOC level (*SOC* system organ class, *N* number of reports).

SOC	N (%)
Gastrointestinal disorders (SOC: 10017947)	4785 (43.66)
Skin and subcutaneous tissue disorders (SOC: 10040785)	2419 (22.07)
General disorders and administration site conditions (SOC: 10018065)	1149 (10.48)
Metabolism and nutrition disorders (SOC: 10027433)	741 (6.76)
Investigations (SOC: 10022891)	631 (5.76)
Blood and lymphatic system disorders (SOC: 10005329)	350 (3.19)
Hepatobiliary disorders (SOC: 10019805)	250 (2.28)
Infections and infestations (SOC: 10021881)	224 (2.04)
Respiratory, thoracic and mediastinal disorders (SOC: 10038738)	188 (1.72)
Cardiac disorders (SOC: 10007541)	99 (0.90)
Injury, poisoning and procedural complications (SOC: 10022117)	43 (0.39)
Psychiatric disorders (SOC: 10037175)	28 (0.26)
Nervous system disorders (SOC: 10029205)	25 (0.23)
Vascular disorders (SOC: 10047065)	18 (0.16)
Renal and urinary disorders (SOC: 10038359)	6 (0.05)
Reproductive system and breast disorders (SOC: 1003860)	3 (0.03)

Among these, the top five SOCs according to the number of reported signals were gastrointestinal disorders (43.66%, 4,785 signals), skin and subcutaneous tissue disorders (22.07%, 2,419 signals), general disorders and administration site conditions (10.48%, 1,149 signals), metabolism and nutrition disorders (6.76%, 741 signals), and investigations (5.76%, 631 signals). Remarkably, more than half of these signals (65.73%) were related to GI and dermatological concerns. Thus, to further investigate lapatinib-related AEs, the researchers conducted a frequency analysis, categorising the PT signals based on the number of reports and their signal strength. In contrast, the less frequently reported systems included cardiac disorders, injury, poisoning and procedural complications, psychiatric disorders, nervous system disorders, vascular disorders, renal and urinary disorders, and reproductive system and breast disorders. None of these rare events were stated in the FDA drug label of lapatinib (U.S. Food and Drug Administration, 2022), except for cardiac signals (Table 4).

### Adverse event signals of preferred terms

Initially, a comprehensive analysis of all signals detected was conducted at the PT level to provide a detailed overview of lapatinib's latest toxicity profile. In total, 155 PT signals were identified to be potentially linked to lapatinib. Among these, 115 signals were significantly disproportionate and met the criteria established by all four algorithms (ROR, PRR, BCPNN, and MGPS), with 57 classified as potentially new PTs (Supplemental Table S1).

The top 50 AEs linked with lapatinib, ranked according to the frequency of reports from highest to lowest, are presented in Table 5. The most predominant AEs included diarrhoea (PT:10012735; 2,444 reports), nausea (PT:10028813; 886 reports), fatigue (PT:10016256; 680 reports), vomiting (PT: 10047700; 623 reports), rash (PT: 10037844; 614 reports), and palmar-plantar erythrodysesthesia syndrome (PPE) (PT:10033553; 401 reports), all of which are listed in lapatinib's FDA drug label (U.S. Food and Drug Administration, 2022). However, the analysis of the study findings revealed several new significant AEs not mentioned on the label (2022). These include weight decreased (PT:10047895; 187 reports), oedema peripheral (PT:10030124; 104 reports), dysphagia (PT:10013950; 96 reports), acne (PT:10000496; 77 reports), cellulitis (PT:10007882; 56 reports), leukopenia (PT:10024384; 50 reports), pleural effusion (PT:10035598; 47 reports), skin ulcer (PT:10040943; 38 reports), blood alkaline phosphatase increased (PT:10059570; 33 reports), ascites (PT:10003445; 31 reports), and pericardial effusion (PT:10034474; 28 reports) (Table 5). Amongst these new signals, only acne and skin ulcer signals showcased medium intensity. It is also worth noting that although AEs like prolonged QT interval, dyspnoea, insomnia, anorexia, alopecia, asthenia, headache, back pain, and arthralgia were mentioned in the drug label (U.S. Food and Drug Administration, 2022), the current study was not able to detect these events using the four detection algorithms. This could be due to several factors, such as the possibility that these events are more prominent when the drug is used in combination with other drugs or the influence of underreporting or missing data in FAERS.

**Table 5.** Detected PT signals of the top 50 adverse events based on the number of lapatinib safety reports (March 2007 to July 2024) (*N* number of cases reporting PT, *ROR* reporting odds ratio, *CI* confidence interval, *PRR* proportional reporting ratio; ***c****^2^* chi-squared, *IC* information component, *IC025* lower limit of 95% CI of the IC, *EBGM* empirical Bayesian geometric mean; *EBGM05* lower limit of 95% CI of EBGM, § new adverse events, *a*: IC025 > 3.0, it indicates a strong signal intensity, *b*: 1.5 < IC025 ≤ 3.0, it indicates a medium signal intensity).

PT	N	ROR (95% two-sided CI)	PRR (c^2^)	IC (IC_025_)	EBGM (EBGM_05_)
Diarrhea	2444	15.30 (14.60 −16.04)	11.21 (23141.02)	3.48 (3.32) ^a^	11.13 (10.70)
Nausea	886	3.34 (3.11–3.58)	3.10 (1295.57)	1.63 (1.52) ^b^	3.09 (2.92)
Fatigue	680	2.61 (2.4–2.83)	2.48 (619.89)	1.31 (1.21)	2.48 (2.32)
Vomiting	623	3.99 (3.68–4.34)	3.78 (1291.21)	1.94 (1.79) ^b^	3.84 (3.59)
Rash	614	4.021 (3.70–4.37)	3.80 (1287.43)	1.92 (1.77) ^b^	3.80 (3.54)
Palmar-Plantar Erythrodysaesthesia Syndrome	401	47.20 (42.64–52.26)	45.03 (16718.63)	5.45 (4.92) ^a^	43.70 (40.14)
Decreased Appetite	332	4.25 (3.81–4.745)	4.13 (788.04)	2.04 (1.83) ^b^	4.12 (3.75)
Dehydration	294	7.18 (6.39–8.07)	6.97 (1497.52)	2.80 (2.49) ^b^	6.94 (6.30)
Dry Skin	198	7.71 (6.69–8.88)	7.55 (1116.40)	2.91 (2.53) ^b^	7.52 (6.68)
Neutropenia	194	4.02 (3.49–4.64)	3.96 (426.59)	1.98 (1.72) ^b^	3.95 (3.50)
Weight Decreased ^§^	187	2.03 (1.75–2.34)	2.01 (94.03)	1.00 (0.87)	2.00 (1.77)
Erythema	185	3.30 (2.86–3.82)	3.25 (287.69)	1.70 (1.47)	3.25 (2.87)
Abdominal Pain	160	2.08 (1.78–2.43)	2.06 (86.85)	1.04 (0.89)	2.06 (1.81)
Stomatitis	154	7.96 (6.78–9.34)	7.83 (908.39)	2.97 (2.53) ^b^	7.81 (6.83)
Skin Fissures	124	25.99 (21.73–31.08)	25.62 (2860.52)	4.66 (3.89) ^a^	25.19 (21.69)
Febrile Neutropenia	106	5.40 (4.45–6.54)	5.34 (369.40)	2.41 (1.99) ^b^	5.33 (4.54)
Oedema Peripheral^§^	104	2.35 (1.93–2.85)	2.33 (77.94)	1.22 (1.00)	2.33 (1.98)
Hypokalemia	97	6.80 (5.56–8.31)	6.73 (466.48)	2.75 (2.25) ^b^	6.71 (5.67)
Dysphagia ^§^	96	2.64 (2.16–3.23)	2.62 (94.69)	1.39 (1.13)	2.62 (2.21)
Skin Exfoliation	91	4.60 (3.74–5.66)	4.56 (249.13)	2.19 (1.78) ^b^	4.55 (3.83)
Skin Discoloration	88	5.89 (4.77–7.27)	5.84 (347.04)	2.54 (2.06) ^b^	5.82 (4.88)
Dyspepsia	89	2.57 (2.08–3.16)	2.55 (82.54)	1.35 (1.09)	2.55 (2.14)
Blood Bilirubin Increased	77	8.16 (6.517–10.22)	8.10 (469.76)	3.01 (2.40) ^b^	8.06 (6.67)
Epistaxis	77	2.95 (2.36–3.70)	2.94 (96.40)	1.55 (1.24)	2.93 (2.43)
Acne ^§^	**77**	**6.04** (**4.82–7.56)**	**5.99** (**314.24)**	**2.58 (2.06) ^b^**	**5.97** (**4.95)**
Alanine Aminotransferase Increased	71	3.40 (2.69–4.30)	3.38 (116.77)	1.76 (1.39)	3.38 (2.78)
Blister	70	3.91 (3.09–4.95)	3.88 (146.96)	1.95 (1.54) ^b^	3.88 (3.18)
Nail Disorder	65	25.59 (20.01–32.74)	25.41 (1474.14)	4.64 (3.63) ^a^	24.98 (20.33)
Jaundice	64	6.50 (5.08–8.31)	6.45 (288.60)	2.68 (2.10) ^b^	6.43 (5.23)
Mouth Ulceration	63	8.74 (6.817–11.207)	8.68 (418.51)	3.11 (2.43) ^b^	8.64 (7.02)
Hepatic Enzyme Increased	56	2.43 (1.871–3.166)	2.43 (45.51)	1.28 (0.98)	2.42 (1.94)
Rash Pruritic	56	3.17 (2.44–4.13)	3.16 (80.45)	1.66 (1.27)	3.15 (2.53)
Cellulitis ^§^	56	3.37 (2.59–4.38)	3.35 (89.91)	1.74 (1.34)	3.34 (2.68)
Paronychia	55	43.35 (33.12–56.73)	43.08 (2154.63)	5.39 (4.12) ^a^	41.86 (33.42)
Mucosal Inflammation	52	6.29 (4.78–8.26)	6.26 (223.61)	2.64 (2.01) ^b^	6.23 (4.96)
Aspartate Aminotransferase Increased	50	2.81 (2.13–3.72)	2.80 (56.18)	1.48 (1.12)	2.80 (2.22)
Leukopenia ^§^	50	3.05 (2.31–4.02)	3.04 (66.18)	1.60 (1.21)	3.03 (2.40)
Skin Toxicity	49	26.30 (19.81–34.91)	26.15 (1139.90)	4.68 (3.53) ^a^	25.69 (20.27)
Dermatitis Acneiform	48	24.86 (18.67–33.09)	24.72 (1051.28)	4.60 (3.46) ^a^	24.32 (19.15)
Pleural Effusion ^§^	47	2.33 (1.75–3.10)	2.32 (33.93)	1.21 (0.91)	2.32 (1.82)
Hepatotoxicity	47	5.36 (4.02–7.14)	5.34 (160.86)	2.41 (1.81)	5.32 (4.18)
Hepatic Function Abnormal	41	3.19 (2.34–4.33)	3.17 (58.87)	1.66 (1.22)	3.17 (2.45)
Ejection Fraction Decreased	38	6.43 (4.67–8.85)	6.41 (167.37)	2.67 (1.94) ^b^	6.38 (4.89)
Skin Ulcer ^§^	**38**	**4.45** (**3.24–6.12)**	**4.44** (**97.51)**	**2.15 (1.56) ^b^**	**4.43** (**3.39)**
Hyperbilirubinemia	35	10.51 (7.53–14.66)	10.47 (288.33)	3.38 (2.42) ^b^	10.40 (7.87)
Blood Alkaline Phosphatase Increased ^§^	33	3.92 (2.78–5.52)	3.91 (68.39)	1.96 (1.39)	3.90 (2.93)
Ascites ^§^	31	3.19 (2.24–4.54)	3.18 (44.21)	1.67 (1.17)	3.18 (2.37)
Pericardial Effusion^§^	28	3.52 (2.43–5.11)	3.51 (47.77)	1.81 (1.25)	3.51 (2.57)
Cardiotoxicity	25	7.23 (4.88–10.72)	7.22 (127.12)	2.84 (1.92) ^b^	7.18 (5.17)
Gastrointestinal Toxicity	19	12.46 (7.93–19.58)	12.43 (186.91)	3.62 (2.31) ^b^	12.34 (8.45)

On the other hand, the top 50 AEs associated with lapatinib, ranked by signal strength from highest to lowest, are illustrated in Table 6. Notably, most of the identified signals exhibited medium intensity (Table 6). However, findings of this study reveal that strong signals were surprisingly more concentrated in nail and skin disorders than in GI signals, such as nail bed bleeding (12 reports, ROR 68.87, 95% CI 38.58-122.94), onychalgia (19 reports, ROR 66.43, 95% CI 41.92-105.26), nail infection (23 reports, ROR 52.95, 95% CI 34.91-80.32), and PPE (401 reports, ROR 47.20, 95% CI 42.64-52.26) (Table 6). Other strong signals include paronychia, skin toxicity, skin fissures, nail disorder, and dermatitis acneiform, all listed in lapatinib's drug label (U.S. Food and Drug Administration, 2022). Interestingly, diarrhoea was the only strong GI signal reported with 2444 cases reports (ROR 15.31, 95% CI 14.60-16.04) (Table 6).

**Table 6.** Detected PT signals of the top 50 adverse events based on the risk signal strength of lapatinib (March 2007 to July 2024) (*N* number of cases reporting PT, *ROR* reporting odds ratio, *CI* confidence interval, *PRR* proportional reporting ratio; ***c****^2^* chi-squared, *IC* information component, *IC025* lower limit of 95% CI of the IC, *EBGM* empirical Bayesian geometric mean; *EBGM05* lower limit of 95% CI of EBGM, § new adverse events, *a*: IC025 > 3.0, it indicates a strong signal intensity, *b*: 1.5 < IC025 ≤3.0, it indicates a medium signal intensity).

PT	N	ROR (95% two-sided CI)	PRR (c^2^)	IC (IC_025_)	EBGM (EBGM_05_)
Nail Bed Bleeding	12	68.87 (38.58–122.94)	68.77 (701.67)	6.04 (3.38) ^a^	65.69 (40.45)
Onychalgia	19	66.43 (41.92–105.26)	66.28 (1106.43)	5.99 (3.78) ^a^	63.42 (43.14)
Nail Infection	23	52.95 (34.91–80.32)	52.81 (1078.41)	5.67 (3.74) ^a^	50.98 (35.98)
Palmar-Plantar Erythrodysaesthesia Syndrome	401	47.20 (42.64–52.26)	45.03 (16718.63)	5.45 (4.92) ^a^	43.70 (40.14)
**Hypocapnia ^§^**	**5**	**46.92** (**19.25–114.35)**	**46.89** (**175.30)**	**5.51 (2.26) ^b^**	**45.45** (**21.57)**
Paronychia	55	43.35 (33.12–56.73)	43.08 (2154.63)	5.39 (4.12) ^a^	41.86 (33.42)
**Milia ^§^**	**5**	**37.44** (**15.40**–**90.99)**	**37.42** (**139.04)**	**5.19 (2.14) ^b^**	**36.50** (**17.36)**
Skin Toxicity	49	26.30 (19.81–34.91)	26.15 (1139.90)	4.68 (3.53) ^a^	25.69 (20.27)
Skin Fissures	124	25.99 (21.73–31.08)	25.62 (2860.52)	4.66 (3.89) ^a^	25.19 (21.69)
Nail Disorder	65	25.59 (20.01–32.74)	25.41 (1474.14)	4.64 (3.63) ^a^	24.98 (20.33)
Dermatitis Acneiform	48	24.86 (18.67–33.09)	24.72 (1051.28)	4.60 (3.46) ^a^	24.32 (19.15)
Onycholysis	11	20.37 (11.23–36.94)	20.34 (180.87)	4.33 (2.39) ^b^	20.07 (12.20)
Palmar Erythema	7	20.36 (9.65–42.94)	20.34 (108.58)	4.33 (2.05) ^b^	20.07 (10.75)
Electrolyte Depletion	3	19.97 (6.39–62.43)	19.97 (36.24)	4.30 (1.38)	19.71 (7.59)
Onychomadesis	21	19.43 (12.62–29.89)	19.38 (343.33)	4.26 (2.77) ^b^	19.14 (13.34)
**In growing Nail ^§^**	**13**	**17.11** (**9.90**–**29.57)**	**17.08** (**178.98)**	**4.08 (2.36) ^b^**	**16.90** (**10.69)**
**Lip Ulceration ^§^**	**6**	**15.74** (**7.04**–**35.19)**	**15.73** (**67.92)**	**3.96 (1.77) ^b^**	**15.57** (**7.94)**
Diarrhea	2444	15.31 (14.60–16.04)	11.21 (23141.02)	3.48 (3.32) ^a^	11.13 (10.70)
**Hepatic Infection ^§^**	**6**	**13.42** (**6.01**–**29.99)**	**13.41** (**56.55)**	**3.73 (1.67) ^b^**	**13.30** (**6.79)**
Onychoclasis	30	13.17 (9.19–18.88)	13.12 (321.19)	3.70 (2.58) ^b^	13.02 (9.63)
Diarrhea Infectious^§^	3	12.90 (4.14–40.21)	12.90 (21.90)	3.68 (1.18)	12.79 (4.94)
Gastrointestinal Toxicity	19	12.46 (7.93–19.58)	12.43 (186.91)	3.62 (2.31) ^b^	12.34 (8.45)
Nasal Septum Perforation^§^	3	12.11 (3.89–37.72)	12.10 (20.29)	3.59 (1.15)	12.01 (4.64)
Blood Bilirubin Unconjugated Increased	3	11.56 (3.71–36.01)	11.55 (19.17)	3.52 (1.13)	11.47 (4.43)
**Cheilitis ^§^**	**21**	**11.22** (**7.30**–**17.24)**	**11.19** (**183.45)**	**3.47 (2.26) ^b^**	**11.11** (**7.76)**
**Nasal Ulcer ^§^**	**6**	**11.12** (**4.98**–**24.83)**	**11.11** (**45.20)**	**3.46 (1.55) ^b^**	**11.03** (**5.63)**
**Chapped Lips ^§^**	**17**	**10.99** (**6.81**–**17.71)**	**10.97** (**143.09)**	**3.44 (2.14) ^b^**	**10.89** (**7.30)**
Radiation Skin Injury ^§^	4	10.55 (3.94–28.21)	10.54 (25.47)	3.39 (1.27)	10.47 (4.60)
Hyperbilirubinemia	35	10.51 (7.53–14.66)	10.47 (288.33)	3.38 (2.42) ^b^	10.40 (7.87)
**Erysipelas ^§^**	**15**	**9.99** (**6.01**–**16.61)**	**9.97** (**111.549)**	**3.31 (1.99) ^b^**	**9.91** (**6.48)**
Electrocardiogram Change	4	9.80 (3.66–26.19)	9.79 (23.23)	3.28 (1.23)	9.73 (4.27)
Enteritis Infectious^§^	5	9.62 (3.99–23.19)	9.62 (30.26)	3.26 (1.35)	9.56 (4.58)
**Feces Pale ^§^**	**7**	**9.52** (**4.53**–**20.02)**	**9.51** (**44.84)**	**3.24 (1.54) ^b^**	**9.45** (**5.08)**
**Lip Pain ^§^**	**9**	**8.88** (**4.61**–**17.10)**	**8.87** (**54.86)**	**3.14 (1.63) ^b^**	**8.82** (**5.09)**
Skin Oedema ^§^	3	8.86 (2.85–27.58)	8.86 (13.71)	3.14 (1.01)	8.80 (3.41)
Enterocolitis Infectious ^§^	3	8.79 (2.83–27.36)	8.79 (13.56)	3.13 (1.01)	8.74 (3.38)
Mouth Ulceration	63	8.74 (6.82–11.21)	8.68 (418.51)	3.11 (2.43) ^b^	8.64 (7.02)
**Jaundice Cholestatic ^§^**	**10**	**8.59** (**4.61**–**16.00)**	**8.58** (**59.26)**	**3.09 (1.66) ^b^**	**8.54** (**5.07)**
Blood Bilirubin Increased	77	8.16 (6.52–10.22)	8.10 (469.76)	3.01 (2.40) ^b^	8.06 (6.67)
Effusion ^§^	4	8.06 (3.02–21.54)	8.06 (18.07)	3.00 (1.12)	8.02 (3.52)
Nail Growth Abnormal ^§^	3	7.80 (2.57–24.88)	7.99 (11.96)	2.99 (0.96)	7.96 (3.08)
Stomatitis	154	7.96 (6.78–9.34)	7.83 (908.39)	2.97 (2.53) ^b^	7.81 (6.83)
Dry Skin	198	7.71 (6.69–8.88)	7.55 (1116.40)	2.91 (2.53) ^b^	7.52 (6.68)
Rash Pustular	19	7.66 (4.88–12.03)	7.65 (102.69)	2.93 (1.86) ^b^	7.61 (5.22)
Blood Electrolytes Abnormal	3	7.42 (2.39–23.08)	7.42 (10.80)	2.88 (0.93)	7.39 (2.86)
Cardiotoxicity	25	7.23 (4.88–10.72)	7.22 (127.12)	2.84 (1.92) ^b^	7.18 (5.17)
Dehydration	294	7.18 (6.39–8.07)	6.97 (1497.52)	2.80 (2.49) ^b^	6.94 (6.30)
Hypokalemia	97	6.8 (5.56–8.31)	6.73 (466.48)	2.75 (2.25) ^b^	6.71 (5.67)
Lip Discoloration ^§^	3	6.75 (2.17–20.99)	6.75 (9.46)	2.75 (0.88)	6.72 (2.60)
Tongue Dry ^§^	3	6.73 (2.16–20.93)	6.73 (9.42)	2.74 (0.88)	6.70 (2.59)

**Figure 2.**
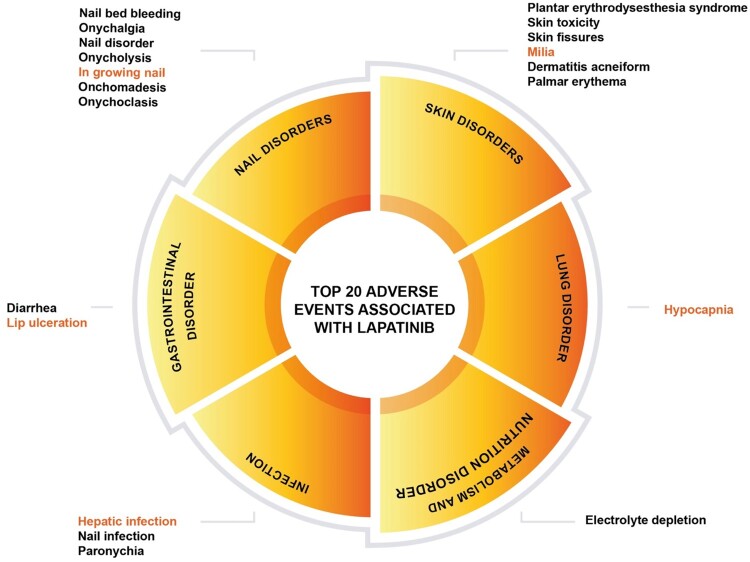
Top 20 significant adverse events associated with lapatinib based on the risk signal strength.

More importantly, this study also highlights 12 potentially new signals of medium intensity that are not included in the FDA label (U.S. Food and Drug Administration, 2022), namely hypocapnia (n = 5), milia (n = 5), in growing nail (n = 13), lip ulceration (n = 6), hepatic infection (n = 6), cheilitis (n = 21), nasal ulcer (n = 6), chapped lips (n = 17), erysipelas (n = 15), faeces pale (n = 7), lip pain (n = 9), and jaundice cholestatic (n = 10). All these suspected new risk signals (total 120 cases) were further evaluated in the case-by-case assessment, as shown in Supplemental Table S2. Patients were reported to have received a lapatinib dose ranging from 1,000–1,500 mg/day, with the majority given 1,250 mg/day. This dosing aligns with the approved therapeutic lapatinib dose for HER-2 positive breast cancer (UpToDate, 2025). Although one case reported a dose of 250 mg/day, we assume that this was likely a reporting error resulting from incomplete documentation of dosing frequency. Further analysis of reporting trends showed that all novel adverse events were reported at a dose of 1,250 mg/day, while certain events, such as milia, ingrowing nail, lip ulceration, cheilitis, nasal ulcer, pale feces, and lip pain, occurred even at lower doses of 1,000 mg/day (Supplemental Table S2). Around 63 cases led to either death, disability, hospitalisation (initial or prolonged), life-threatening situations, situations requiring medical interventions, or other serious events, while the remaining did not have any specified outcome. In addition, based on the causality assessment using the Naranjo algorithm, the majority of the suspected new cases were classified as ‘possible’ or ‘probable’, with the majority being ‘possible’ AEs related to lapatinib (n = 100 vs. n = 20, respectively). Other AEs include, but are not limited to, ascites, mouth haemorrhage, peripheral oedema, effusion, and pericardial effusion (Supplemental Table S1). Figure 2 summarises the top 20 strong AEs associated with lapatinib.

## Discussion

Adverse events can profoundly impact patients, leading to compromised quality of life (QoL) and, in severe cases, death, thereby increasing both clinical and economic burdens (Khalil & Huang, 2020; Sultana et al., 2013). Consequently, post-marketing surveillance is crucial for identifying serious drug-related safety concerns, which can ultimately help mitigate life-threatening outcomes and enhance QoL. Unlike clinical trials with limitations in detecting the full range of AEs, such as small sample sizes, strict inclusion criteria, and short follow-up periods, real-world studies offer a wider interpretation of AEs potentially associated with medications. This is particularly valuable for identifying rare AEs that may only become evident through long-term surveillance. Currently, several pieces of evidence on lapatinib are mostly related to clinical trials and literature reviews focusing on its mechanism of action and pharmacokinetics. More recently, while few studies have used FAERS data to depict its AEs in the real world, these studies have predominantly addressed HER-2 inhibitors as a class (Bao et al., 2024; Barbieri et al., 2022; Tang et al., 2025; Waliany et al., 2023; Wittayanukorn et al., 2017), with limited emphasis on lapatinib alone. In addition, although one study has analysed the real-world safety of lapatinib (Xie et al., 2020), there remains a lack of pharmacovigilance studies that have comprehensively investigated the signal strengths of AEs linked with lapatinib via established signal detection algorithms. Hence, the present study fills this gap by providing a more detailed analysis of AEs associated with lapatinib through a disproportionate analysis of real-world FAERS data.

This study shows that lapatinib-induced AEs significantly affected women (77.90%, n = 29,650) more than men, as lapatinib was primarily indicated for treating BC. This finding aligns with prior FAERS studies on lapatinib or HER-2 inhibitors (Barbieri et al., 2022; Tang et al., 2025). Lapatinib is FDA-approved for adults with locally advanced BC whose tumours overexpress HER-2 and who have failed prior chemotherapy (Agency). Numerous studies have also demonstrated its efficacy in both metastatic (Cameron et al., 2008; Kaufman et al., 2009; Toi et al., 2009; Wang et al., 2021) and early BC in neoadjuvant settings (Baselga et al., 2012; de Azambuja et al., 2014). In terms of age distribution, the majority were adults aged 18 years and above (56.74%, n = 21,697), which is consistent with existing clinical trials and epidemiological BC data (Baselga et al., 2012; Blackwell et al., 2010; ‘Breast cancer incidence (invasive) statistics,’ 2024; Wang et al., 2021), as well as real-world studies on lapatinib/HER-2 inhibitors (Barbieri et al., 2022; Tang et al., 2025). The risk of developing BC increases with age (‘Breast Cancer Risk Factors,’ 2024), and current trends indicate a steady rise in BC incidence rates from age 25–29, with the highest rates between ages 50 and 65 (‘Breast cancer incidence (invasive) statistics,’ 2024; ‘Breast Cancer Risk Factors,’ 2024). Furthermore, this study identified reports regarding the off-label use of lapatinib in patients under 18 years. A similar finding was reported in a recent study conducted by Tang et al. (2025), in which three cases involved paediatric patients. Although no clinical trials have been conducted in this population, we suggest that these reports may likely stem from experimental or more personalised management in rare HER-2-related cancers in children (Tang et al., 2025). Given the unique metabolism and drug response mechanisms in this population, it is essential for healthcare providers like pharmacists to perform vigilant safety monitoring to minimise potential drug toxicities whilst ensuring optimal drug efficacy, especially with long-term lapatinib therapy. Additionally, given that adverse event reporting via FAERS is voluntary, it is inevitable that reports may have data entry errors or missing information, and hence, such limitations must be considered whilst interpreting the present study findings.

Throughout the study period, 10,959 lapatinib signals involving 16 organ systems were mined, with the majority being medium intensity. Notably, based on the disproportionality analysis, SOCs with higher occurrence rates and signals often pertain to GI disorders (like diarrhoea, nausea, and vomiting) (43.66%) and skin and subcutaneous tissue disorders (like rash and PPE) (22.07%). These findings align with prior safety trials of lapatinib (Baselga et al., 2012; de Azambuja et al., 2014; Hurvitz & Kakkar, 2012; Yang et al., 2018) and recent FAERS studies on HER-2 inhibitors (Bao et al., 2024; Barbieri et al., 2022; Tang et al., 2025). However, the present study suggests that skin and subcutaneous tissue disorders, especially nail disorders, are more strongly associated with lapatinib based on the detection algorithms, which aligns with a recent pharmacovigilance study on HER-2 inhibitors (Tang et al., 2025). According to Tang et al. (2025), nail deformities such as nail bed bleeding, onychalgia, and nail bed disorder were among the top 20 AEs associated with lapatinib based on the ROR (Tang et al., 2025). Furthermore, many AEs listed on the lapatinib drug specification, such as stomatitis, dyspepsia, mucosal inflammation, paronychia, dermatitis acneiform, and pneumonitis (U.S. Food and Drug Administration, 2022), were all significantly detected. Interestingly, in addition to the commonly reported systems, the current study found several potentially new affected systems, including but not limited to infections and infestations, vascular disorders, and nervous system disorders, which is in agreement with previous studies (Bao et al., 2024; Barbieri et al., 2022). Unfortunately, most AEs in these categories are not stated in the drug label of lapatinib nor emphasised in trials, highlighting the need for further research to establish a potential causal relation between these AEs and lapatinib. This is especially important for the newly identified top 50 medium-intensity significant signals, such as hypocapnia, milia, in growing nail, lip ulceration, hepatic infection, cheilitis, nasal ulcer, chapped lips, erysipelas, faeces pale, lip pain, and cholestatic jaundice.

GI concerns were the most frequently reported lapatinib-related adverse event (43.66%, 4,785 signals). However, only diarrhoea exhibited a strong signal, indicating a significant correlation. Other common GI signals, like nausea and vomiting, were of medium intensity. These findings align with the existing literature (Bao et al., 2024; Barbieri et al., 2022; Baselga et al., 2012; Blackwell et al., 2009; Gomez et al., 2008; Sardesai & Storniolo, 2015; Yang et al., 2018), suggesting that, unlike other GI AEs, lapatinib primarily induces diarrhoea. For instance, findings from the NeoALTTO trial reported a higher prevalence of grade 3 diarrhoea with lapatinib alone (23.4%) and lapatinib plus trastuzumab (21.1%) compared to only trastuzumab (2%) (Baselga et al., 2012). Similarly, a study combining lapatinib with letrozole showed an almost eight-fold increase in diarrhoea (68%), while nausea and vomiting increased by only about 10% in the combination arm (Schwartzberg et al., 2010). Several mechanisms have been proposed to explain lapatinib-induced diarrhoea, including ErbB1 expression in the GI mucosa, altered chloride secretion, and changes in gut microflora (Raja Sharin et al., 2022). However, the exact mechanism still remains unclear. Additionally, some of the top new GI PTs related to lapatinib that have not been previously reported in other literature or FDA labelling, including dysphagia, oral pain, and ascites, necessitate further investigation. On the contrary, while the present study identified a strong association between diarrhoea and lapatinib, this contradicts findings reported by Tang et al. (2025). In this study, unlike skin and nail disorders, gastrointestinal signals were surprisingly not among the top 20 signals most strongly associated with lapatinib (Tang et al., 2025). Nevertheless, given the negative impact of such AEs on patients’ QoL, it is therefore critical to adapt effective strategies like rehydration or the use of anti-diarrheal (Crown et al., 2008; Moy & Goss, 2007) or anti-emetic agents (Jordan et al., 2014) to manage lapatinib-induced diarrhoea and nausea/vomiting, respectively.

This study also showed that despite the high frequency of GI signals, in terms of both signal frequency and strength, lapatinib appeared to be significantly more likely to cause skin and subcutaneous tissue toxicities. This aligns with the current evidence (Abdel-Rahman & Fouad, 2015; Lacouture et al., 2009; Schwartzberg et al., 2010; Tang et al., 2025; Xu et al., 2011). A phase III trial on the combination of lapatinib and letrozole for metastatic BC showed that the incidence of rash was six times higher in the combination arm (46% vs. 8%) (Schwartzberg et al., 2010). Another study found that 50% of patients on lapatinib developed a rash, with 35% occurring within six weeks (Sonnenblick et al., 2016). Additionally, PPE (hand-foot syndrome) was largely reported and strongly linked with lapatinib in this study. However, Geyer et al. reported no statistically significant difference in PPE incidences between patients receiving both lapatinib and capecitabine versus capecitabine alone (Geyer et al., 2006). While this shows that PPE may be more attributable to capecitabine, reported PPE cases in this study likely stem from combination therapy (U.S. Food and Drug Administration, 2022). Nevertheless, the findings of this study suggest that lapatinib might have a stronger correlation with dermatological adverse events compared to GI events, except for diarrhoea. Notably, some studies show that ErbB1/EGFR is expressed in the skin and plays a crucial role in the normal physiological functioning of skin tissues (Abdullah et al., 2012; King et al., 1990; Lacouture, 2006; Lynch et al., 2007; Rodeck et al., 1997; Segaert & Van Cutsem, 2005). Hence, inhibiting EGFR in the dermis and epidermis via lapatinib may result in disruption of keratinocyte proliferation and migration, induction of keratinocyte apoptosis (King et al., 1990; Lacouture, 2006; Lynch et al., 2007; Rodeck et al., 1997; Segaert & Van Cutsem, 2005), and influx of inflammatory mediators (Abdullah et al., 2012; Chatsiproios, 2010). Given that these skin reactions can affect QoL and therapy compliance, skin monitoring and pro-active interventions such as topical emollients, corticosteroids, etc. are deemed necessary to minimise such reactions (Kwakman et al., 2020).

It is also evident that several less-reported nail-related PT signals, such as paronychia, were strongly associated with lapatinib, aligning with current evidence (Tang et al., 2025; Tischer et al., 2017). According to existing studies, nail changes occur 2–6 months post-therapy with EGFR inhibitors and may persist after therapy cessation (Galimont-Collen et al., 2007). However, the exact mechanism and risk factors behind these nail disorders are poorly understood, warranting further investigation. Thus, it is crucial to monitor such conditions to minimise the risk of infection and other complications. Interestingly, other less-reported signals in this study were related to cardiac adverse events. These findings align with current research suggesting that, unlike other HER-2 inhibitors, lapatinib is less frequently associated with cardiac toxicities, even in combination therapy (Bao et al., 2024; Barbieri et al., 2022; Waliany et al., 2023; Wittayanukorn et al., 2017). Furthermore, cardiac AEs linked to lapatinib were mainly asymptomatic, reversible, non-cumulative, and not dose-dependent, unlike anthracyclines- or Herceptin-induced cardiac effects (Moy & Goss, 2007; Perez et al., 2008), making it less concerning.

Indeed, the analysis of risk signal strengths has unveiled several potentially new AEs not documented in lapatinib's drug label (U.S. Food and Drug Administration, 2022), which were deemed possibly related to lapatinib according to the Naranjo algorithm. While potential causal relationships cannot be established using a case-by-case analysis alone, findings from this analysis facilitates the prioritisation of clinically relevant risk signals for further signal assessments via clinical studies such as prospective cohort studies (Cutroneo et al., 2024). Several novel adverse events were detected pertaining to respiratory disorders like hypocapnia, infections including liver infection, skin and nail conditions such as milia and ingrowing nails, and oral disorders like lip ulceration. The exact mechanisms behind these events remain unclear. However, given that patients were reported to have both interstitial lung disease (ILD) and hypocapnia concurrently (Supplemental Table S2), lapatinib-induced ILD could be a plausible explanation for hypocapnia indirectly. ILD is characterised by inflammation and/or fibrosis in the lung interstitium, impairing the lungs’ ability to efficiently exchange gases (Althobiani et al., 2024; Pippalapalli & Lumb, 2023), and thereby triggering compensatory hyperventilation, potentially leading to hypocapnia (Pippalapalli & Lumb, 2023). Meanwhile, lapatinib-induced hepatic infections may stem from cancer patients’ compromised immunity and acute liver toxicities, as evidenced by elevated hepatic enzymes. On the other hand, ingrown nails and milia, which are tiny, white, superficial cysts due to entrapped keratin (Berk & Bayliss, 2008), are also linked to the disruption of keratinocyte migration (King et al., 1990; Lacouture, 2006; Lynch et al., 2007; Rodeck et al., 1997; Segaert & Van Cutsem, 2005). Lip ulceration may be related to lapatinib-induced mucositis due to mucosal irritation in the lips; however, its underlying mechanism is not yet fully understood (Galimont-Collen et al., 2007). Lastly, it is worth noting that in addition to the top new AEs associated with lapatinib, the present study also detected other new safety signals that may be serious, such as peripheral oedema, dysphagia, leukopenia, pericardial effusion, and pulmonary thrombosis. Further investigation using robust clinical data is needed to validate the potential causal relationship with lapatinib. Nevertheless, while these events may be less reported, their serious nature and impact on patients’ QoL warrant regular monitoring of patients’ symptoms, vitals, and blood tests to determine abnormalities like elevated white blood cells or C-reactive protein, likely indicating infection, liver function tests, and respiratory function, such as arterial blood gases, to monitor for hypocapnia. While patients experiencing lip ulceration or oral mucositis must be managed with appropriate topical and systemic analgesics or local anaesthetics.

Notably, with the rapid advancement in the field of artificial intelligence (AI), machine learning (ML) integrated with FAERS data holds tremendous potential to significantly enhance risk prediction modelling methods. ML algorithms have the capability to recognise patterns and then predict the likelihood of an adverse event linked to a particular drug by analysing factors like patient demographics and drug characteristics (Ali & Aoun, 2023). In addition, the incorporation of ML with FAERS data has the ability to provide real-time risk prediction estimates as soon as new data becomes available (Mitchell & Rabbi, 2023), resulting in more proactive safety surveillance compared to traditional analytical methods. ML algorithms have the capability to recognise patterns and then predict the likelihood of an adverse event linked to a particular drug by analysing factors like patient demographics and drug characteristics (Ali & Aoun, 2023). In addition, the incorporation of ML with FAERS data has the ability to provide real-time risk prediction estimates as soon as new data becomes available (Mitchell & Rabbi, 2023), resulting in more proactive safety surveillance compared to traditional analytical methods. However, the adoption and interpretation of complex machine learning models remain challenging, particularly in addressing issues like bias and potential confounders, which must be carefully accounted for to prevent any skewed or inaccurate predictions (Ali & Aoun, 2023).(Ali & Aoun, 2023). Moreover, while integrating AI and ML could revolutionise drug safety monitoring, analysis of FAERS data using ML techniques still requires thorough validation, domain expertise, and careful consideration of the inherent constraints of the data.

It is clear that this study has certain limitations, some of which are due to the inherent nature of FAERS and OpenVigil. Firstly, although disproportionality analysis was used to identify statistically significant risk signals, it cannot be used as a standalone method to establish a drug-related risk or causal link between drug exposure and AEs (Cutroneo et al., 2024; Elisabetta et al., 2012). Therefore, in this study, a case-by-case analysis of the top new suspected AEs of moderate signal intensity was conducted to complement the disproportionality analysis findings by validating detected signals potentially directing future clinical studies to confirm a causal relation with lapatinib (Cutroneo et al., 2024). Although causality was assessed independently, a key limitation of the case-by-case analysis is that the causal assessment was solely based on the limited information provided in FAERS reports via OpenVigil, and that subjective judgment could not be fully eliminated. Furthermore, critical variables for a comprehensive causality evaluation, such as patients’ laboratory findings, comorbidities, and drug rechallenge data, were not available, which may potentially lead to underestimation or overestimation of the causality strength. Secondly, the FAERS database has several inherent biases, such as notoriety bias, confounding bias due to co-existing comorbidities or concomitant medications, and the potential for underreporting and reporting bias, which may negatively influence the accuracy of our results (Elisabetta et al., 2012). Furthermore, the FAERS database only captures information on spontaneously reported cases of adverse events and does not include exposure data. Consequently, this study was limited to evaluating disproportionality and could not estimate dose-dependent risks of developing significant adverse events due to the lack of exposure data. Thirdly, given these associated biases, this study could not compare the safety profile of lapatinib when used in combination therapy. More robust clinical and retrospective studies are needed to compare the safety profile of lapatinib alone versus in combination with other anti-cancer agents. Fourthly, several FAERS reports had missing data (e.g. data on gender and age). Lastly, OpenVigil may have some technical limitations, such as ambiguity in the outcome labelled as ‘others,’ which requires further clarification.

## Conclusion

This study used FAERS data via OpenVigil to mine and analyse adverse event signals associated with lapatinib. The findings reveal numerous risk signals, with GI and dermatological disorders being the most frequently reported events. Interestingly, dermatological events, particularly nail and skin disorders, were more strongly associated with lapatinib, while diarrhoea emerged as the only strong GI signal. This underscores the need for heightened monitoring of cutaneous side effects during treatment. In contrast, cardiac events were less commonly found to be linked with lapatinib. Moreover, this study identified potentially concerning new signals among the top 50 strongest signals, including but not limited to hypocapnia, lip ulceration, and hepatic infection, which require further signal assessments and clinical studies beyond statistical associations presented in this study to confirm potential causal relationships with lapatinib. Despite certain limitations, this study provides additional insights into the adverse events associated with lapatinib in the real-world setting that can potentially complement ongoing safety reviews by the FDA and pharmaceutical industries.

## Supplementary Material

Supplementary Table S2.docx

Supplementary Table S1.docx

## Data Availability

The datasets generated during and/or analysed in this study are available in OpenVigil 2.1 MedDRA v24 or from the corresponding author upon reasonable request.
