# Supplementary material for: Signal mining and safety profile analysis of lapatinib: a pharmacovigilance analysis of the FDA Adverse Event Reporting System (FAERS) database
Source: J Pharm Policy Pract. 2026 Jan 13;19(1):2611182. doi: 10.1080/20523211.2025.2611182 (PMC12802520; doi:10.1080/20523211.2025.2611182)
Supplement: Supplementary Table S2.docx [file JPPP_A_2611182_SM5798.docx]

**Supplementary Table S2.** Case-by-case assessment of top new adverse events potentially related to lapatinib using OpenVigil (*Causality assessment was evaluated at the time of the present analysis using Naranjo Adverse Drug Reaction Probability Scale in which the potential causal relation between each ADE and lapatinib can be classified as: high probable (score ≥ 9), probable (scores 5–8), possible (scores 1–4), or doubtful (score ≤ 0))

| **New Adverse Events** | **Case No.** | **Case ID** | **Level of Seriousness** | **Year** | **Sex** | **Reporter Country** | **Age** | | **Concomitant Medications** | **Reported**  **Dose** | **Other Reported Adverse Events** | **Causality Assessment (Score) *** |
| --- | --- | --- | --- | --- | --- | --- | --- | --- | --- | --- | --- | --- |
| **Hypocapnia** | 1 | 7250199 | Hospitalization - Initial or Prolonged | 2011 | Female | Japan | 69 Years | | Capecitabine | 1250 mg/day | - Hypercapnia - Interstitial Lung Disease - Dyspnea - Hypoxia | Possible (2) |
|  | 2 | 7086459 | Hospitalization - Initial or Prolonged | 2009 | Female | Japan | Not Specified | | Capecitabine | 1250 mg/day | - Pyrexia - Dyspnea - Hypercapnia - Hypoxia - Interstitial Lung Disease - Malaise | Possible (1) |
|  | 3 | 7214492 | - Life-Threatening - Hospitalization- Initial or Prolonged - Disability - Other | 2009 | Female | Unites States | 68 Years | | Paclitaxel | 1500 mg/day | - Tachypnoea - Lung Neoplasm - Neuropathy Peripheral - Pleuritic Pain - Pyrexia - Rales - Anemia - Chest Pain - Cough - Dyspnea - Fatigue - Hypercapnia - Hypoxia - Interstitial Lung Disease - Lung Infiltration | Possible (1) |
|  | 4 | 6585930 | Hospitalization - Initial or Prolonged | 2008 | Female | Canada | 58 Years | | Capecitabine  Hydrochlorothiazide  Irbesartan | 1250 mg/day | - Hypercapnia - Cough - Dehydration - Diarrhea - Vomiting - Syncope - Pleural Effusion - Hypoxia - Dyspnea | Possible (1) |
|  | 5 | 6695389 | Hospitalization - Initial or Prolonged | 2008 | Female | Unites States | 56 Years | | Zoledronic Acid  Trastuzumab | Not Specified | - Tachypnoea - Disease Progression - Cough - Dyspnea - Hypercapnia - Hypoxia - Interstitial Lung Disease - Metastatic Neoplasm - Pneumonitis - Pyrexia - Rales | Possible (1) |
| **Milia** | 1 | 7240568 | Not Specified | 2010 | Female | United States | 57 Years | | None | 5 tablets/day, dose unknown | - Nausea - Sleep Disorder - Skin Infection - Rash Pustular - Rash Pruritic - Rash - Acne - Condition Aggravated - Fatigue - Insomnia - Morbid Thoughts | Possible (4) |
|  | 2 | 6611347 | Not Specified | 2008 | Female | United States | Not Specified | | None | Not specified | - Insomnia - Muscle Spasms - Muscle Tightness - Sensation Of Heaviness - Sensation Of Pressure - Back Pain - Feeling Abnormal | Possible (4) |
|  | 3 | 8558270 | Not Specified | 2012 | Female | United States | Not Specified | | Trastuzumab | 1000 mg/day | - Pruritus - Acne - Laceration - Purulent Discharge - Rash | Possible (4) |
|  | 4 | 7939664 | Not Specified | 2011 | Female | United States | 65 Years | | Capecitabine  Trastuzumab | 250 mg five times/day | - Laceration - Diarrhea - Rash - Fatigue - Pruritus - Retching - Disease Progression - Dry Skin | Possible (4) |
|  | 5 | 7939717 | Not Specified | 2011 | Female | United States | 54 Years | | Trastuzumab | 1000 mg/day | - Dehydration - Frequent Bowel Movements - Vomiting - Rash - Nausea - Malaise - Decreased Appetite - Weight Decreased | Possible (4) |
| **In growing Nail** | 1 | 9283281 | Not Specified | 2014 | Female | United States | 65 Years | | Trastuzumab  Warfarin  Baclofen  Esomeprazole  Felodipine  Trazodone  Vitamin C  Pyridoxine  Vitamin B12 | 1000 mg/day | - Skin Discoloration - Diarrhea - Onycholysis - Local Swelling - Skin Disorder | Probable (5) |
|  | 2 | 8526314 | - Death - Hospitalization - Initial or Prolonged | 2012 | Female | United Kingdom | 43 Years | | Capecitabine  Dexamethasone  Carboplatin  Etoposide | 1250 mg/day | - Food Craving - Aggression - Mouth Ulceration - Urinary Tract Disorder - Dyspepsia - Headache - Pyuria - Treatment Noncompliance - Fatigue - Dysarthria - Drooling - Nausea - Neutrophilia - Nail Infection - Diarrhea - Tooth Disorder - Vomiting - Drug Ineffective - Breast Cancer Metastatic - Hypoesthesia - Oedema - Confusional State - Balance Disorder - Pollakiuria | Probable (5) |
|  | 3 | 17360633 | - Hospitalization - Initial or Prolonged - Other | 2021 | Female | Not specified | Not Specified | | None | 1000 mg/day | - Feeling Abnormal - Hypersensitivity - Malaise - Nail Injury - Limb Injury - Metastases To Central Nervous System - Illness - Dysstasia - Eating Disorder - Diarrhea - Abdominal Pain Upper | Probable (5) |
|  | 4 | 8644014 | Other | 2012 | Female | United States | Not Specified | | Capecitabine | 1000 mg/day | - Nausea - Blister - Treatment Noncompliance - Diarrhea - Palmar-Plantar Erythrodysaesthesia Syndrome - Paronychia - Inappropriate Schedule of Drug Administration | Possible (4) |
|  | 5 | 8557976 | Not Specified | 2013 | Female | United States | 62 Years | | Famotidine  Mupirocin  Lisinopril  Cephalexin  Simvastatin  Aspirin  Levothyroxine  Diltiazem  Zinc  Vitamin C | 1000 mg/day | - Nasopharyngitis - Nail Disorder - Nail Infection - Cough - Epistaxis - Dysphagia - Diarrhea - Nasal Ulcer | Possible (4) |
|  | 6 | 6439966 | Not Specified | 2007 | Female | United States | Not Specified | | None | Not specified | None | Possible (4) |
|  | 7 | 7939994 | Not Specified | 2011 | Female | United States | 76 Years | | Capecitabine  Alprazolam | 5 tablets/day, dose unknown | - Nail Infection - Rash | Possible (4) |
|  | 8 | 6439969 | Not Specified | 2007 | Female | United States | Not Specified | | None | Not specified | None | Possible (4) |
|  | 9 | 8354367 | Hospitalization - Initial or Prolonged | 2012 | Female | Australia | Not Specified | | Capecitabine | Not specified | Paronychia | Possible (4) |
|  | 10 | 8557916 | Not Specified | 2012 | Female | United States | 53 Years | | Capecitabine  Omeprazole  Midodrine  Carvedilol | 5 tablets/day, dose unknown | - Diarrhea - Skin Chapped | Possible (4) |
|  | 11 | 8530065 | Other | 2013 | Female | Canada | 55 Years | | Capecitabine  Escitalopram  Verapamil  Lorazepam | 1250 mg/day | - Breast Cancer Metastatic - Skin Fissures - Skin Exfoliation - Pain In Extremity - Dry Skin - Gait Disturbance - Palmar-Plantar Erythrodysaesthesia Syndrome - Erythema - Dyspepsia - Skin Atrophy | Possible (4) |
|  | 12 | 6876846 | Not Specified | 2009 | Female | United States | 53 years | | Capecitabine  Omeprazole  Midodrine  Carvedilol | 5 tablets/day, dose unknown | Skin Chapped | Possible (4) |
|  | 13 | 6876847 | Not Specified | 2009 | Female | United States | Not Specified | | Not specified | 5 tablets/day, dose unknown | None | Possible (4) |
| **Lip Ulceration** | 1 | 7240551 | Not Specified | 2010 | Female | United States | 55 Years | | Capecitabine | 1250 mg/day | - Arthralgia - Chapped Lips - Fatigue - Palmar-Plantar Erythrodysaesthesia Syndrome - Skin Ulcer - Stomatitis | Possible (1) |
|  | 2 | 7350513 | Other | 2010 | Female | United States | 54 Years | | Capecitabine  Trastuzumab  Valsartan  Letrozole  Fluoxetine | 1250 mg/day | - Abdominal Pain - Blister - Diarrhea - Glossodynia - Erythema - Mouth Ulceration - Pain In Extremity - Palmar-Plantar Erythrodysaesthesia Syndrome - Pharyngeal Erythema - Skin Chapped - Skin Exfoliation - Stomatitis - Swollen Tongue | Possible (1) |
|  | 3 | 20278921 | - Death - Hospitalization - Initial or Prolonged - Other | 2022 | Female | China | 42 Years | | Capecitabine | 1250 mg/day | - Cough - Pneumonia - Dizziness - Malignant Neoplasm Progression - Dehydration - Chest Discomfort - Breast Cancer - Breast Cancer Recurrent - Confusional State - Multiple Organ Dysfunction Syndrome - Headache - Blood Uric Acid Increased | Possible (4) |
|  | 4 | 8892288 | Other | 2012 | Female | United States | 55 Years | | Capecitabine | 1250 mg/day | - Skin Ulcer - Stomatitis - Arthralgia - Chapped Lips - Fatigue - Palmar-Plantar Erythrodysaesthesia Syndrome | Possible (1) |
|  | 5 | 10685336 | Not Specified | 2015 | Not Specified | United States | Not Specified | | Capecitabine  Ramipril | 250 mg, frequency unknown | - Oropharyngeal Pain - Pharyngeal Erythema | Possible (4) |
|  | 6 | 10159848 | Not Specified | 2014 | Female | United States | 36 years | | Valaciclovir  Trastuzumab  Omeprazole  Aspirin  Lorazepam | 1000 mg/day | - Rash - Pruritus - Rash Generalized - Stomatitis - Cheilitis - Food Poisoning - Acne - Diarrhea - Off Label Use - Drug Ineffective - Nasal Dryness - Tongue Ulceration - Oral Pain - Epistaxis - Tongue Eruption - Eating Disorder | Possible (1) |
| **Hepatic Infection** | 1 | 11683495 | Death | 2015 | Female | Not Specified | 35 Years | | None | 5 tablets/day, dose unknown | None | Possible (4) |
|  | 2 | 9657359 | Death | 2013 | Female | India | 35 Years | | None | 4 tablets/day, dose unknown | - Abdominal Pain Upper - Jaundice - Myocardial Infarction | Possible (4) |
|  | 3 | 11658701 | Death | 2015 | Female | Not Specified | 43 Years | | None | 4 tablets/day, dose unknown | Death | Possible (4) |
|  | 4 | 11648972 | Death | 2015 | Female | Not Specified | 47 Years | | None | 250 mg, frequency unknown | Death | Possible (4) |
|  | 5 | 9675886 | Death | 2013 | Female | India | 58 Years | | None | 250 mg/day | Chest Pain | Possible (4) |
|  | 6 | 11156684 | - Other - Death | 2015 | Female | India | 58 Years | | None | 250 mg five times/day | - Jaundice - Bedridden - Death - Food Intolerance | Possible (4) |
| **Cheilitis** | 1 | 8761247 | Disability | 2012 | Female | Japan | 52 Years | | Capecitabine | 1250 mg/day | None | Possible (4) |
|  | 2 | 10159598 | Not Specified | 2014 | Female | United States | 52 Years | | Trastuzumab  Pamidronic Acid  Levothyroxine  Glimepiride  Bupropion  Losartan | 5 tablets/day, dose unknown | - Malaise - Nasopharyngitis - Dry Skin - Lip Oedema | Possible (1) |
|  | 3 | 9283156 | Not Specified | 2013 | Female | United States | Not Specified | | None | 250 mg, frequency unknown | Lip Pain | Possible (4) |
|  | 4 | 6439992 | Other | 2008 | Female | United States | 52 Years | | Exemestane  Zoledronate Zoledronic Acid  Salmeterol Xinafoate Fluticasone Propionate Salmeterol Fluticasone  Warfarin  Theophylline  Calcium  Ergocalciferol Cholecalciferol Vitamin D | 5 tablets/day, dose unknown | - Hepatic Enzyme Increased - Folliculitis - Dyspnea - Exertional - Dyspnea - Cough - Skin Ulcer - Weight Decreased - Rash Papular - Pruritus | Possible (1) |
|  | 5 | 8351791 | Other | 2012 | Female | Australia | 54 Years | | Capecitabine | Not Specified | - Pain In Extremity - Palmar-Plantar Erythrodysaesthesia Syndrome - Erythema | Possible (4) |
|  | 6 | 6440057 | Not Specified | 2007 | Female | United States | 56 Years | | Citalopram  Bupropion  Gabapentin | 1250 mg/day | - Chapped Lips - Dry Mouth | Possible (1) |
|  | 7 | 8875837 | Other | 2013 | Female | Japan | 61 Years | | Capecitabine  Trastuzumab | Not Specified | - Hypokalaemia - Rash - Paronychia - Diarrhea | Possible (1) |
|  | 8 | 6350798 | Not Specified | 2007 | Female | United States | 52 Years | | Trastuzumab  Pamidronate Pamidronic Acid  Levothyroxine  Glimepiride  Bupropion | 5 tablets/day, dose unknown | - Dry Skin - Lip Oedema - Lip Oedema | Possible (1) |
|  | 9 | 7939582 | Not Specified | 2011 | Female | United States | 49 Years | | Capecitabine  Omeprazole  Naproxen  Venlafaxine  Levothyroxine | 1250 mg/day | - Skin Disorder - Nausea - Chest Discomfort - Diarrhea | Possible (1) |
|  | 10 | 7717157 | Hospitalization - Initial or Prolonged | 2010 | Female | Canada | Not Specified | | Dexamethasone  Capecitabine | 1250 mg/day | - Condition Aggravated - Dehydration - Diarrhea - Dysgeusia - Increased Appetite - Rash - Skin Ulcer - Thermal Burn - Vomiting | Possible (4) |
|  | 11 | 10655444 | Not Specified | 2015 | Female | United States | Not Specified | | None | 1250 mg/day | - Condition Aggravated - Dehydration - Diarrhea - Dysgeusia - Increased Appetite - Rash - Skin Ulcer - Thermal Burn - Vomiting | Possible (4) |
|  | 12 | 7350409 | Not Specified | 2010 | Female | United States | 50 Years | | Diltiazem  Esomeprazole  Levothyroxine  Levetiracetam | Not Specified | - Muscular Weakness - Feeling Hot - Erythema - Dizziness - Skin Exfoliation | Possible (4) |
|  | 13 | 9283382 | Not Specified | 2013 | Female | United States | 78 Years | | Capecitabine  Gemfibrozil  Lisinopril  Metformin  Trastuzumab | 5 tablets/day, dose unknown | - Muscular Weakness - Feeling Hot - Erythema - Dizziness - Skin Exfoliation | Possible (1) |
|  | 14 | 7046731 | Not Specified | 2009 | Female | United States | 48 Years | | Fondaparinux Sodium  Fentanyl | 4 tablets/day, dose unknown | - Dry Mouth - Vomiting - Diarrhea | Possible (1) |
|  | 15 | 14470559 | Not Specified | 2018 | Female | United States | 82 Years | | None | 1000 mg/day | - Dry Mouth - Vomiting - Diarrhea | Possible (1) |
|  | 16 | 6351082 | Not Specified | 2007 | Female | United States | 49 Years | | Capecitabine  Warfarin  Levothyroxine  Venlafaxine  Fentanyl  Oxycodone | 1250 mg/day | - Nausea - Diarrhea - Skin Chapped | Possible (1) |
|  | 17 | 15240542 | Not Specified | 2018 | Female | United States | Not Specified | | None | 1250 mg/day | Rash | Possible (4) |
|  | 18 | 11159750 | Not Specified | 2015 | Female | United States | Not Specified | | None | 1250 mg/day | - Decreased Appetite - Dysgeusia - Fatigue - Gastroesophageal Reflux Disease - Stomatitis - Vomiting - Weight Decreased | Possible (4) |
|  | 19 | 10159842 | Not Specified | 2014 | Female | United States | 46 Years | | Capecitabine | 1250 mg/day | - Diarrhea - Weight Decreased - Adverse Drug Reaction | Possible (4) |
|  | 20 | 16372153 | Other | 2019 | Female | Canada | 69 Years | | Trastuzumab | 1000 mg/day | - Diarrhea - Dry Skin - Ejection Fraction Decreased - Brain Neoplasm Malignant - Nausea - Skin Irritation - Abdominal Pain Upper - Asthenia - Fatigue - Stomatitis | Possible (1) |
|  | 21 | 10159848 | Not Specified | 2014 | Female | United States | 36 Years | | Valaciclovir  Trastuzumab  Omeprazole  Aspirin  Lorazepam | 1000 mg/day | - Lip Ulceration - Eating Disorder - Food Poisoning - Stomatitis - Acne - Diarrhea - Off Label Use - Drug Ineffective - Rash Generalized - Tongue Ulceration - Oral Pain - Epistaxis - Tongue Eruption - Pruritus - Nasal Dryness - Rash | Possible (4) |
| **Nasal Ulcer** | 1 | 8557922 | Not Specified | 2012 | Female | United States | 52 Years | | Warfarin | 5 tablets/day, dose unknown | - Dry Skin - Diarrhea - Laceration - Rash - Dermatitis Acneiform - Epistaxis - Cough - Lip Discoloration | Possible (4) |
|  | 2 | 8557976 | Not Specified | 2013 | Female | United States | 62 Years | | Famotidine  Mupirocin  Lisinopril  Cephalexin  Simvastatin  Aspirin  Levothyroxine  Diltiazem  Zinc  Vitamin C | 1000 mg/day | - Nail Infection - Epistaxis - Dysphagia - Nail Disorder - Diarrhea - In growing Nail - Nasopharyngitis - Cough | Possible (4) |
|  | 3 | 10159642 | Not Specified | 2014 | Female | United States | Not Specified | | None | 1250 mg/day | - Epistaxis - Skin Atrophy - Hepatic Pain - Diarrhea - Pain - Nausea | Possible (4) |
|  | 4 | 6351011 | Not Specified | 2007 | Female | United States | 59 Years | | None | Not Specified | - Paresthesia - Scab - Diarrhea - Dyspepsia | Possible (4) |
|  | 5 | 7240581 | Not Specified | 2010 | Female | United States | 58 Years | | Celecoxib  Rosuvastatin  Venlafaxine  Cyclobenzaprine  Ibandronate  Ceftriaxone | 1000 mg/day | - Epistaxis - Lip Discoloration - Rash - Skin Laceration - Cough - Diarrhea - Dry Skin | Possible (4) |
|  | 6 | 7350452 | Not Specified | 2010 | Female | United States | 52 Years | | Warfarin | 5 tablets/day, dose unknown | - Epistaxis - Lip Discoloration - Rash - Skin Laceration - Cough - Diarrhea - Dry Skin | Possible (4) |
| **Chapped Lips** | 1 | 7352166 | Not Specified | 2010 | Female | Japan | 61 Years | | Capecitabine | 1250 mg/day | - Decreased Appetite - Diarrhea - Dry Skin - Dyspepsia | Probable (5) |
|  | 2 | 6440076 | Not Specified | 2007 | Female | United States | 55 Years | | Citalopram  Bupropion  Gabapentin  Zoledronic Acid | 250 mg five times/day | - Nausea - Fatigue - Pruritus Generalized | Possible (1) |
|  | 3 | 13025242 | Not Specified | 2017 | Female | United States | 71 Years | | None | 1250 mg/day | - Neuralgia - Nausea - Fatigue - Diarrhea | Possible (4) |
|  | 4 | 9364854 | Hospitalization - Initial or Prolonged | 2013 | Female | Canada | Not Specified | | Capecitabine  Hydrocortisone  Levothyroxine  Oxycodone  Acetaminophen  Pantoprazole  Oxazepam  Loperamide  Tinzaparin | 1250 mg/day | - Asthenia - Vomiting - Fatigue - Lip Pain - Dyspnea - Diarrhea - Skin Fissures - Constipation - Phlebitis - Pain In Extremity - Nausea - Breast Cancer Metastatic | Possible (1) |
|  | 5 | 7939729 | Not Specified | 2011 | Female | United States | Not Specified | | None | 1250 mg/day | - Dry Skin - Skin Exfoliation - Stomatitis - Pain In Extremity | Possible (1) |
|  | 6 | 7940022 | Not Specified | 2011 | Female | United States | Not Specified | | Trastuzumab  Fulvestrant | 1250 mg/day | - Decreased Appetite - Diarrhea - Increased Appetite - Weight Decreased - Fatigue - Dry Mouth | Possible (1) |
|  | 7 | 8892288 | Other | 2012 | Female | United States | 55 Years | | Capecitabine | 1250 mg/day | - Stomatitis - Arthralgia - Fatigue - Lip Ulceration - Palmar-Plantar Erythrodysaesthesia Syndrome - Skin Ulcer | Possible (4) |
|  | 8 | 6440057 | Not Specified | 2007 | Female | United States | 56 Years | | Citalopram  Bupropion  Gabapentin | 1250 mg/day | - Cheilitis - Dry Mouth | Possible (1) |
|  | 9 | 7240551 | Not Specified | 2010 | Female | United States | 55 Years | | Capecitabine | 1250 mg/day | - Arthralgia - Stomatitis - Skin Ulcer - Palmar-Plantar Erythrodysaesthesia Syndrome - Lip Ulceration - Fatigue | Possible (4) |
|  | 10 | 7904848 | Other | 2011 | Female | Ireland | Not Specified | | Capecitabine | 1250 mg/day | - Laceration - Skin Fissures - Disease Progression | Possible (4) |
|  | 11 | 7290757 | Other | 2010 | Female | United States | 49 Years | | Capecitabine  Loratadine  Diphenhydramine  Simvastatin | 5 tablets five times per day, dose unknown | - Diarrhea - Nausea - Gastroenteritis - Feces Pale - Dry Skin - Vomiting - Urinary Tract Infection - Stomatitis - Pyrexia - Oral Pain | Possible (4) |
|  | 12 | 9283352 | Not Specified | 2013 | Female | United States | 54 Years | | None | 250 mg, frequency unknown | - Glossodynia - Lip Pain - Dysgeusia | Possible (4) |
|  | 13 | 8100208 | Other | 2011 | Female | Ireland | Not Specified | | Capecitabine | 1250 mg/day | - Disease Progression - Laceration - Skin Fissures | Possible (4) |
|  | 14 | 8487630 | Other | 2012 | Female | France | 46 Years | | None | Not Specified | - Diarrhea - Actinomycosis - Erythema - Gait Disturbance - Skin Plaque - Rash | Possible (4) |
|  | 15 | 6350944 | Not Specified | 2007 | Female | United States | 63 Years | | Capecitabine  Ondansetron  Acetaminophen Hydrocodone | 1250 mg/day | - Restlessness - Oral Pain - Nausea - Diarrhea - Rash | Possible (4) |
|  | 16 | 6350945 | Not Specified | 2007 | Female | United States | Not Specified | | None | Not Specified | - Diarrhea - Lip Dry | Possible (1) |
|  | 17 | 8557941 | Not Specified | 2012 | Female | United States | 62 Years | | Trastuzumab  Fulvestrant | 1250 mg/day | - Increased Appetite - Fatigue - Weight Decreased - Diarrhea - Decreased Appetite - Dry Mouth | Possible (1) |
| **Erysipelas** | 1 | 6643459 | Hospitalization - Initial or Prolonged | 2008 | Female | Denmark | 33 Years | | Capecitabine | 1250 mg/day | Lymphoedema | Probable (6) |
|  | 2 | 7034651 | Hospitalization - Initial or Prolonged | 2009 | Female | Russian Federation | Not Specified | | Trastuzumab | Not Specified | - Pyrexia - Erythema - Swelling Face - Rash - Pain | Possible (3) |
|  | 3 | 6589600 | Hospitalization - Initial or Prolonged | 2008 | Female | Germany | 45 Years | | None | 1500 mg/day | Pyrexia | Probable (6) |
|  | 4 | 6511509 | Other | 2007 | Female | Luxembourg | 74 Years | | Capecitabine | 1250 mg/day | - Oedema - Skin Fissures - Rash Erythematous - Skin Ulcer | Probable (6) |
|  | 5 | 6356694 | Hospitalization - Initial or Prolonged | 2007 | Female | Italy | 47 Years | | Capecitabine | 1250 mg/day | - Skin Oedema - Rash | Probable (6) |
|  | 6 | 6755063 | Hospitalization - Initial or Prolonged | 2009 | Female | Germany | 41 Years | | Trastuzumab | 1500 mg/day | - Eczema - Erythema - Inflammation - Pyrexia - Skin Toxicity - Swelling - Cellulitis | Possible (3) |
|  | 7 | 6322705 | Hospitalization - Initial or Prolonged | 2007 | Female | Germany | 65 Years | | Calcium Chloride  Magnesium Sulfate | 1500 mg/day | - Streptococcal Sepsis - Anemia - Bunion - Infection - Hypokalemia - Hyperthermia - Paronychia - Sinus Tachycardia - Lymphoedema | Possible (2) |
|  | 8 | 7802146 | Hospitalization - Initial or Prolonged | 2011 | Female | Japan | 62 Years | | Capecitabine  Zoledronic Acid  Amlodipine  Valsartan  Entecavir | 1250 mg/day | None | Probable (6) |
|  | 9 | 6617011 | Hospitalization - Initial or Prolonged | 2010 | Female | Denmark | Not Specified | | Capecitabine | 1250 mg/day | - Oedema Peripheral - Pruritus - Vasculitis - Endocarditis | Possible (4) |
|  | 10 | 6791471 | Hospitalization - Initial or Prolonged | 2008 | Female | Italy | 64 Years | | Capecitabine | 1250 mg/day | - Infection - Palmar-Plantar Erythrodysaesthesia Syndrome | Probable (5) |
|  | 11 | 6988948 | Hospitalization - Initial or Prolonged | 2009 | Female | Germany | Not Specified | | Trastuzumab  Paclitaxel | 1500 mg/day | - Swelling - Erythema - Leukocytosis - Nausea - Pyrexia | Possible (3) |
|  | 12 | 6384817 | Hospitalization - Initial or Prolonged | 2007 | Female | Slovakia (Slovak Republic) | | 55 Years | Capecitabine | 1250 mg/day | None | Probable (6) |
|  | 13 | 6511488 | Hospitalization - Initial or Prolonged | 2008 | Female | Germany | 62 Years | | None | 1500 mg/day | None | Probable (6) |
|  | 14 | 7980590 | Other | 2011 | Female | Poland | 56 Years | | Capecitabine | Not Specified | Palmar-Plantar Erythrodysaesthesia Syndrome | Probable (5) |
|  | 15 | 6653341 | Hospitalization - Initial or Prolonged | 2008 | Female | Finland | 59 Years | | Capecitabine | 1250 mg/day | Palmar-Plantar Erythrodysaesthesia Syndrome | Probable (5) |
| **Feces Pale** | 1 | 6747915 | Other | 2008 | Female | South Africa | 47 Years | | Trastuzumab  Paclitaxel  Esomeprazole | 1000 mg/day | - Abdominal Pain Upper - Alanine Aminotransferase Increased - Diarrhea - Hyperbilirubinemia - Perihepatic Discomfort | Probable (5) |
|  | 2 | 6605238 | Hospitalization - Initial or Prolonged | 2008 | Female | United Kingdom | 63 Years | | Capecitabine  Lansoprazole  Atenolol  Zoledronate  Acetaminophen/ Codeine  Dexamethasone | 1250 mg/day | - Fecal Incontinence - Hemorrhoids - Blood Potassium Decreased - Herpes Zoster - Malaise - Pyrexia - Rash - Urinary Incontinence - Urinary Tract Infection - Dysuria | Possible (4) |
|  | 3 | 16424817 | Not Specified | 2019 | Female | United States | 69 Years | | None | Not Specified | - Abdominal Discomfort - Diarrhea | Possible (3) |
|  | 4 | 8558201 | Not Specified | 2015 | Not Specified | United States | Not Specified | | None | 250 mg, frequency unknown | - Rhinorrhea - Visual Impairment - Fatigue - Lacrimation Increased | Probable (5) |
|  | 5 | 6722365 | Hospitalization - Initial or Prolonged | 2008 | Female | United States | 48 Years | | Capecitabine | 1250 mg/day | - Abdominal Pain Upper - Chromaturia - Dyspnea - Hepatic Mass - Malaise - Ocular Icterus - Yellow Skin | Possible (1) |
|  | 6 | 7290757 | Other | 2010 | Female | United States | 49 Years | | Capecitabine  Loratadine  Diphenhydramine  Simvastatin | 5 tablets five times/day, dose unknown | - Gastroenteritis - Chapped Lips - Diarrhea - Dry Skin - Nausea - Oral Pain - Pyrexia - Stomatitis - Urinary Tract Infection - Vomiting | Possible (3) |
|  | 7 | 8558089 | Not Specified | 2012 | Female | United States | 64 Years | | Capecitabine  Trastuzumab | 1250 mg/day | - Diarrhea - Abdominal Pain Upper | Possible (4) |
| **Lip Pain** | 1 | 9283156 | Not Specified | 2013 | Female | United States | Not Specified | | None | 250 mg, frequency unknown | Cheilitis | Possible (1) |
|  | 2 | 8557938 | Not Specified | 2012 | Female | United States | 70 Years | | Metformin  Glyburide | 1000 mg/day | - Hot Flush - Hair Disorder - Fatigue - Disease Progression - Facial Pain - Pruritus - Pain In Extremity - Lacrimation Increased - Asthenia - Liver Disorder - Toothache - Eye Pain - Rash - Ocular Hyperemia - Medication Error - Erythema - Malaise - Pain Of Skin - Oedema Peripheral - Poor Quality Sleep - Local Swelling - Dysstasia | Possible (4) |
|  | 3 | 16565038 | - Death - Other | 2019 | Female | Canada | Not Specified | | Capecitabine | 1250 mg/day | - Balance Disorder - Diarrhea - Drug Ineffective - Dry Mouth - General Physical Health Deterioration - Hypersensitivity - Lip Swelling - Memory Impairment - Metastases To Central Nervous System - Nausea - Vomiting | Possible (1) |
|  | 4 | 11408828 | Not Specified | 2015 | Female | United States | Not Specified | | None | Not Specified | - Chills - Diarrhea - Feeding Disorder - Hyperhidrosis - Nausea | Possible (1) |
|  | 5 | 8422887 | Other | 2012 | Female | Australia | 64 Years | | Capecitabine | 1250 mg/day | - Weight Decreased - Fatigue - Skin Chapped - Palmar-Plantar Erythrodysaesthesia Syndrome - Nausea - Skin Reaction - Malaise - Oral Pain - Erythema | Possible (2) |
|  | 6 | 9283352 | Not Specified | 2013 | Female | United States | 54 Years | | None | 250 mg, frequency unknown | - Chapped Lips - Glossodynia - Dysgeusia | Possible (1) |
|  | 7 | 9364854 | Hospitalization - Initial or Prolonged | 2013 | Female | Canada | Not Specified | | Capecitabine  Hydrocortisone  Levothyroxine  Oxycodone  Acetaminophen  Pantoprazole  Oxazepam  Loperamide  Tinzaparin | 1250 mg/day | - Nausea - Phlebitis - Breast Cancer Metastatic - Chapped Lips - Vomiting - Constipation - Asthenia - Fatigue - Dyspnea - Pain In Extremity - Diarrhea - Skin Fissures | Possible (1) |
|  | 8 | 7350392 | Not Specified | 2010 | Female | United States | 70 Years | | None | 5 tablets/day, dose unknown | - Pruritus - Diarrhea - Back Pain | Possible (4) |
|  | 9 | 7940006 | Not Specified | 2011 | Female | United States | 70 Years | | Metformin  Glyburide | 1000 mg/day | - Liver Disorder - Pruritus - Pain In Extremity - Hot Flush - Dysstasia - Oedema Peripheral - Pain Of Skin - Medication Error - Asthenia - Erythema - Fatigue - Lacrimation Increased - Poor Quality Sleep - Hair Disorder - Rash - Malaise - Pain - Ocular Hyperemia - Toothache - Local Swelling - Eye Pain | Possible (4) |
| **Jaundice Cholestatic** | 1 | 6387895 | - Hospitalization - Initial or Prolonged - Other | 2007 | Female | Australia | 56 Years | | Capecitabine | 1250 mg/day | - Bile Duct Stone - Cholangitis - Left Ventricular Dysfunction | Possible (2) |
|  | 2 | 6737380 | Death | 2008 | Female | United Kingdom | 65 Years | | Capecitabine  Nystatin  Cefuroxime  Metronidazole  Lactulose  Morphine  Midazolam | 1250 mg/day | Dehydration | Possible (3) |
|  | 3 | 7645314 | - Death - Hospitalization - Initial or Prolonged | 2010 | Female | Argentina | Not Specified | | Capecitabine | 1250 mg/day | - Pancreatic Carcinoma - Abdominal Pain | Probable (5) |
|  | 4 | 7142166 | Death | 2010 | Female | Japan | 52 Years | | Capecitabine  Oxycodone | 1250 mg/day | Hepatic Function Abnormal | Possible (2) |
|  | 5 | 8181605 | Other | 2011 | Female | France | 54 Years | | None | 1250 mg/day | Hepatocellular Injury | Probable (6) |
|  | 6 | 6399482 | - Hospitalization - Initial or Prolonged - Other | 2007 | Female | Australia | 56 Years | | Capecitabine | Not Specified | - Cholangitis - Bile Duct Stone - Left Ventricular Dysfunction | Possible (2) |
|  | 7 | 8193417 | - Death - Hospitalization - Initial or Prolonged - Other | 2011 | Female | Japan | 51 Years | | Capecitabine | Not Specified | - Hypercalcemia - Blood Bilirubin Increased - Superior Vena Cava Syndrome - Death - Face Oedema - Dyspnea | Probable (6) |
|  | 8 | 20119834 | - Death - Hospitalization - Initial or Prolonged | 2021 | Female | China | 38 Years | | Capecitabine  Potassium Chloride  Hydrotalcite  Oxycodone  Meperidine  Tramadol  Phloroglucinol | 250 mg five times/day | - Asthenia - Malignant Neoplasm Progression - Decreased Appetite - Scleral Discoloration - Abdominal Distension - Abdominal Pain - Skin Discoloration - Breast Cancer - Cholecystitis | Possible (2) |
|  | 9 | 7207648 | Hospitalization - Initial or Prolonged | 2009 | Female | Argentina | 36 Years | | Capecitabine | 1250 mg/day | - Bile Duct Stent Insertion - Pancreatic Mass | Possible (2) |
|  | 10 | 7800093 | - Hospitalization - Initial or Prolonged - Other | 2011 | Female | Japan | 51 Years | | Capecitabine  Zoledronic Acid | 1500 mg/day | None | Probable (6) |
