# Supplementary material for: Signal mining and safety profile analysis of lapatinib: a pharmacovigilance analysis of the FDA Adverse Event Reporting System (FAERS) database
Source: J Pharm Policy Pract. 2026 Jan 13;19(1):2611182. doi: 10.1080/20523211.2025.2611182 (PMC12802520; doi:10.1080/20523211.2025.2611182)
Supplement: Supplementary Table S1.docx [file JPPP_A_2611182_SM5797.docx]

**Supplementary Table S1.** List of adverse events associated with Lapatinib at the Systems Organ Class (SOC) and Preferred Terms (PTs) level from FAERS (*Arranged based on cases count*) [*N* number of cases reporting PT, *ROR* reporting odds ratio, *CI* confidence interval, *PRR* proportional reporting ratio; ***χ****^2^* chi-squared, *IC* information component, *IC025* lower limit of 95% CI of the IC, *EBGM* empirical Bayesian geometric mean; *EBGM05* lower limit of 95% CI of EBGM, *statistically significant adverse events according to ROR, PRR, BCPNN, and MGPS, § new adverse events]

| **SOC** | **PT** | **N** | **ROR**  **(95% two-sided CI)** | **PRR**  **(χ^2^)** | **IC**  **(IC_025_)** | **EBGM**  **(EBGM_05_)** |
| --- | --- | --- | --- | --- | --- | --- |
| **Gastrointestinal Disorders**  [includes 32 PTs] | Diarrhea* | 2444 | 15.305 ( 14.601 - 16.042) | 11.212 ( 23141.023) | 3.477 (3.317) | 11.133 (10.703) |
|  | Nausea* | 886 | 3.338 ( 3.114 - 3.579 ) | 3.096 (1295.573) | 1.628 (1.519) | 3.091 (2.916) |
|  | Vomiting* | 623 | 3.996 ( 3.683 - 4.336 ) | 3.778 (1291.217) | 1.942 (1.789) | 3.841 (3.588) |
|  | Abdominal Pain | 160 | 2.08 ( 1.779 - 2.432 ) | 2.06 (86.847) | 1.041 (0.890) | 2.058 (1.806) |
|  | Stomatitis* | 154 | 7.959 ( 6.784 - 9.338 ) | 7.834 (908.389) | 2.965 (2.527) | 7.810 (6.832) |
|  | Dyspepsia* | 89 | 2.567 ( 2.083 - 3.164 ) | 2.551 (82.543) | 1.349 (1.095) | 2.548 (2.139) |
|  | **Dysphagia ^§^*** | **96** | **2.637 ( 2.156 - 3.225 )** | **2.619 (94.688)** | **1.387 (1.134)** | **2.616 (2.210)** |
|  | Mouth Ulceration* | 63 | 8.741 ( 6.817 - 11.207 ) | 8.684 (418.508) | 3.111 (2.426) | 8.638 (7.016) |
|  | Gastrointestinal Toxicity* | 19 | 12.459 ( 7.928 - 19.58 ) | 12.433 (186.909) | 3.625 (2.306) | 12.336 (8.450) |
|  | **Ascites ^§^*** | **31** | **3.191 ( 2.242 - 4.542 )** | **3.183 (44.211)** | **1.668 (1.172)** | **3.178 (2.365)** |
|  | **Oral Pain ^§^*** | **40** | **5.06 ( 3.707 - 6.907 )** | **5.041 (125.24)** | **2.330 (1.707)** | **5.027 (3.875)** |
|  | Gastritis | 18 | 2.076 ( 1.307 - 3.299 ) | 2.074 (8.964) | 1.051 (0.662) | 2.073 (1.407) |
|  | **Enteritis ^§^*** | **10** | **4.897 ( 2.631 - 9.114 )** | **4.892 (27.11)** | **2.287 (1.229)** | **4.879 (2.901)** |
|  | **Cheilitis ^§^*** | **21** | **11.217 ( 7.297 - 17.241 )** | **11.191 (183.451)** | **3.474 (2.260)** | **11.113 (7.756)** |
|  | Diarrhea Hemorrhagic ^§^ | 8 | 2.61 ( 1.304 - 5.223 ) | 2.608 (6.395) | 1.381 (0.690) | 2.605 (1.458) |
|  | **Chapped Lips ^§^*** | **17** | **10.985 ( 6.814 - 17.711 )** | **10.966 (143.094)** | **3.445 (2.137)** | **10.890 (7.303)** |
|  | Glossodynia ^§^ | 16 | 2.711 ( 1.659 - 4.429 ) | 2.708 (15.548) | 1.435 (0.879) | 2.705 (1.793) |
|  | **Mouth Hemorrhage ^§^*** | **9** | **3.686 ( 1.916 - 7.093 )** | **3.683 (14.976)** | **1.878 (0.976)** | **3.676 (2.126)** |
|  | Lip Dry ^§^ | 8 | 3.294 ( 1.645 ; 6.594 ) | 3.292 (10.553) | 1.717 (0.857) | 3.286 (1.839) |
|  | **Lip Ulceration ^§^*** | **6** | **15.737 ( 7.037 ; 35.191 )** | **15.727 (67.924)** | **3.961 (1.771)** | **15.568 (7.939)** |
|  | **Lip Pain ^§^*** | **9** | **8.875 ( 4.607 - 17.097 )** | **8.866 (54.857)** | **3.141 (1.630)** | **8.818 (5.095)** |
|  | **Feces Pale ^§^*** | **7** | **9.517 ( 4.525 - 20.019 )** | **9.51 (44.841)** | **3.241 (1.541)** | **9.455 (5.075)** |
|  | **Anorectal Discomfort ^§^*** | **9** | **5.187 ( 2.695 - 9.984 )** | **5.183 (26.25)** | **2.370 (1.231)** | **5.168 (2.988)** |
|  | Proctalgia ^§^ | 6 | 3.205 ( 1.438 - 7.142 ) | 3.203 (7.008) | 1.677 (0.753) | 3.199 (1.636) |
|  | Oral Mucosal Blistering | 6 | 2.656 ( 1.192 - 5.918 ) | 2.655 (4.637) | 1.407 (0.631) | 2.652 (1.356) |
|  | Ileus Paralytic ^§^ | 5 | 3.396 ( 1.412 - 8.171 ) | 3.395 (6.208) | 1.761 (0.732) | 3.389 (1.626) |
|  | Tongue Ulceration* | 6 | 5.012 ( 2.248 - 11.174 ) | 5.009 (15.397) | 2.320 (1.040) | 4.993 (2.553) |
|  | Gastrointestinal Sounds Abnormal ^§^ | 5 | 3.005 ( 1.249 - 7.228 ) | 3.004 (4.82) | 1.585 (0.659) | 3.000 (1.439) |
|  | Esophageal Stenosis ^§^ | 4 | 3.999 ( 1.499 - 10.673 ) | 3.998 (6.226) | 1.996 (0.748) | 3.990 (1.755) |
|  | Lip Blister | 4 | 4.067 ( 1.524 - 10.853 ) | 4.065 (6.415) | 2.020 (0.757) | 4.057 (1.784) |
|  | **Lip Discoloration ^§^*** | **3** | **6.751 ( 2.171 - 20.991 )** | **6.749 (9.457)** | **2.749 (0.884)** | **6.722 (2.602)** |
|  | **Tongue Dry ^§^*** | **3** | **6.73 ( 2.164 - 20.926 )** | **6.728 (9.416)** | **2.744 (0.882)** | **6.698 (2.592)** |
| **Skin and Subcutaneous Tissue Disorders**  [includes 38 PTs] | Rash* | 614 | 4.021 ( 3.704 - 4.366 ) | 3.804 (1287.43) | 1.925 (1.773) | 3.797 (3.544) |
|  | Palmar-Plantar Erythrodysesthesia Syndrome* | 401 | 47.203 ( 42.637 -52.258) | 45.034 (16718.63) | 5.450 (4.922) | 43.702 (40.136) |
|  | Dry Skin* | 198 | 7.706 ( 6.691 8.876 ) | 7.551 (1116.4) | 2.910 (2.527) | 7.517 (6.679) |
|  | Erythema* | 185 | 3.303 ( 2.855 - 3.821 ) | 3.253 (287.687) | 1.699 (1.469) | 3.248 (2.875) |
|  | Skin Fissures* | 124 | 25.986 ( 21.731 -31.076 ) | 25.624 ( 2860.516) | 4.655 (3.893) | 25.194 (21.693) |
|  | Skin Exfoliation* | 91 | 4.598 ( 3.739 - 5.655 ) | 4.56 (249.127) | 2.185 (1.777) | 4.548 (3.825) |
|  | Blister* | 70 | 3.908 ( 3.088 - 4.946 ) | 3.884 (146.964) | 1.955 (1.545) | 3.877 (3.183) |
|  | Skin Discoloration* | 88 | 5.886 ( 4.769 - 7.265 ) | 5.836 (347.044) | 2.540 (2.058) | 5.817 (4.877) |
|  | **Acne ^§^*** | **77** | **6.035 ( 4.82 - 7.556 )** | **5.989 (314.241)** | **2.577 (2.058)** | **5.969 (4.945)** |
|  | Nail Disorder* | 65 | 25.593 ( 20.009 -32.736 ) | 25.406 (1474.141) | 4.643 (3.630) | 24.984 (20.333) |
|  | Skin Toxicity* | 49 | 26.297 ( 19.809 - 34.91 ) | 26.152 (1139.9) | 4.683 (3.528) | 25.690 (20.268) |
|  | Dermatitis Acneiform* | 48 | 24.857 ( 18.672 - 33.09 ) | 24.723 (1051.279) | 4.604 (3.459) | 24.324 (19.145) |
|  | Rash Pruritic * | 56 | 3.173 ( 2.439 - 4.127 ) | 3.158 (80.446) | 1.657 (1.274) | 3.154 (2.531) |
|  | **Skin Ulcer ^§^*** | **38** | **4.451 ( 3.235 - 6.124 )** | **4.436 (97.507)** | **2.146 (1.560)** | **4.426 (3.389)** |
|  | Skin Disorder | 31 | 2.541 ( 1.785 - 3.616 ) | 2.535 (27.294) | 1.341 (0.942) | 2.532 (1.885) |
|  | Rash Erythematous | 28 | 2.043 ( 1.41 - 2.962 ) | 2.04 (13.823) | 1.027 (0.709) | 2.038 (1.494) |
|  | Skin Lesion | 19 | 2.011 ( 1.282 - 3.155 ) | 2.009 (8.64) | 1.006 (0.641) | 2.008 (1.377) |
|  | Onychoclasis* | 30 | 13.168 ( 9.186 - 18.876 ) | 13.125 (321.191) | 3.702 (2.583) | 13.016 (9.630) |
|  | Dermatitis | 16 | 2.637 ( 1.614 - 4.308 ) | 2.634 (14.604) | 1.395 (0.854) | 2.631 (1.745) |
|  | **In growing Nail ^§^*** | **13** | **17.108 ( 9.898 - 29.571 )** | **17.084 (178.982)** | **4.079 (2.360)** | **16.896 (10.689)** |
|  | Onychalgia* | 19 | 66.426 (41.919 -105.261) | 66.281 (1106.434) | 5.987 (3.778) | 63.416 (43.144) |
|  | Onychomadesis* | 21 | 19.425 (12.622 - 29.893 ) | 19.379 (343.328) | 4.258 (2.767) | 19.136 (13.341) |
|  | Rash Papular* | 21 | 3.579 ( 2.331 - 5.495 ) | 3.573 (36.308) | 1.834 (1.195) | 3.566 (2.491) |
|  | Pain of Skin* | 16 | 3.363 ( 2.058 - 5.494 ) | 3.358 (24.145) | 1.745 (1.068) | 3.353 (2.223) |
|  | Onycholysis* | 11 | 20.366 ( 11.228 - 36.943) | 20.341 (180.867) | 4.327 (2.386) | 20.073 (12.196) |
|  | Skin Hemorrhage ^§^ | 13 | 3.135 ( 1.819 - 5.405 ) | 3.132 (16.763) | 1.645 (0.954) | 3.127 (1.983) |
|  | **Pigmentation Disorder ^§^*** | **10** | **4.507 ( 2.422 - 8.389 )** | **4.503 (23.793)** | **2.168 (1.165)** | **4.492 (2.672)** |
|  | Nail Bed Bleeding* | 12 | 68.868 ( 38.578 - 122.94) | 68.773 (701.665) | 6.038 (3.382) | 65.692 (40.451) |
|  | Nail Discoloration* | 8 | 5.744 ( 2.868 - 11.506 ) | 5.74 (26.646) | 2.516 (1.256) | 5.721 (3.199) |
|  | **Skin Atrophy ^§^*** | **8** | **4.393 ( 2.194 - 8.796 )** | **4.39 (17.636)** | **2.131 (1.064)** | **4.379 (2.450)** |
|  | Skin Hypertrophy ^§^ | 4 | 4.466 ( 1.673 - 11.92 ) | 4.464 (7.544) | 2.154 (0.807) | 4.450 (1.957) |
|  | Yellow Skin | 6 | 3.588 ( 1.61 - 7.998 ) | 3.587 (8.734) | 1.840 (0.826) | 3.580 (1.831) |
|  | Palmar Erythema* | 7 | 20.357 ( 9.652 - 42.937 ) | 20.341 (108.581) | 4.327 (2.052) | 20.073 (10.750) |
|  | **Skin Wrinkling ^§^*** | **6** | **6.534 ( 2.929 - 14.574 )** | **6.53 (22.737)** | **2.701 (1.211)** | **6.501 (3.323)** |
|  | Skin Tightness ^§^ | 5 | 3.926 ( 1.632 - 9.447 ) | 3.925 (8.146) | 1.969 (0.818) | 3.914 (1.877) |
|  | **Milia ^§^*** | **5** | **37.437 ( 15.403 -90.987 )** | **37.415 (139.043)** | **5.190 (2.135)** | **36.496 (17.359)** |
|  | **Skin Oedema ^§^*** | **3** | **8.863 ( 2.848 - 27.582 )** | **8.86 (13.71)** | **3.138 (1.008)** | **8.805 (3.405)** |
|  | **Nail Growth Abnormal ^§^*** | **3** | **7.996 ( 2.57 - 24.876 )** | **7.994 (11.96)** | **2.992 (0.962)** | **7.955 (3.078)** |
| **General Disorders and Administration Site Conditions**  [includes 8 PTs] | Fatigue* | 680 | 2.612 ( 2.415 - 2.825 ) | 2.483 (619.894 ) | 1.311 (1.212) | 2.481 (2.323) |
|  | Pyrexia* | 294 | 2.66 ( 2.368 - 2.989 ) | 2.603 (292.018) | 1.379 (1.227) | 2.600 (2.359) |
|  | **Oedema Peripheral ^§^*** | **104** | **2.347 ( 1.934 - 2.848 )** | **2.33 (77.938)** | **1.219 (1.005)** | **2.328 (2.000)** |
|  | Mucosal Inflammation* | 52 | 6.287 ( 4.784 - 8.263 ) | 6.255 (223.605) | 2.640 (2.009) | 6.233 (4.959) |
|  | **Crepitations ^§^*** | **6** | **5.874 ( 2.634 - 13.101 )** | **5.871 (19.541)** | **2.549 (1.143)** | **5.851 (2.991)** |
|  | Necrosis ^§^ | 6 | 2.799 ( 1.256 - 6.237 ) | 2.798 (5.241) | 1.483 (0.665) | 2.795 (1.429) |
|  | **Effusion ^§^*** | **4** | **8.06 ( 3.016 - 21.54 )** | **8.057 (18.067)** | **3.003 (1.124)** | **8.018 (3.523)** |
|  | **Axillary Pain ^§^*** | **3** | **6.13 ( 1.972 - 19.057 )** | **6.128 (8.219)** | **2.610 (0.840)** | **6.107 (2.364)** |
| **Metabolism and Nutrition Disorders**  [includes 6 PTs] | Dehydration* | 294 | 7.182 ( 6.392 - 8.071 ) | 6.97 (1497.516) | 2.795 (2.487) | 6.941 (6.296) |
|  | Decreased Appetite* | 332 | 4.251 ( 3.809 - 4.745 ) | 4.125 (788.044) | 2.041 (1.829) | 4.116 (3.754) |
|  | Hypokalaemia* | 97 | 6.8 ( 5.564 - 8.31 ) | 6.734 (466.482) | 2.746 (2.247) | 6.707 (5.671) |
|  | Electrolyte Imbalance | 9 | 2.432 ( 1.264 - 4.679 ) | 2.431 (6.208) | 1.280 (0.665) | 2.428 (1.405) |
|  | **Hyperuricemia ^§^*** | **6** | **5.149 ( 2.309 - 11.48 )** | **5.146 (16.051)** | **2.359 (1.058)** | **5.131 (2.623)** |
|  | Electrolyte Depletion* | 3 | 19.973 ( 6.39 - 62.426 ) | 19.966 (36.236) | 4.301 (1.376) | 19.708 (7.595) |
| **Investigations**  [includes 17 PTs] | Weight Decreased ^§^ | 187 | 2.027 ( 1.753 - 2.343 ) | 2.005 (94.034) | 1.002 (0.867) | 2.003 (1.774) |
|  | Alanine Aminotransferase Increased* | 71 | 3.402 ( 2.693 - 4.298 ) | 3.382 (116.768) | 1.756 (1.389) | 3.377 (2.777) |
|  | Blood Bilirubin Increased* | 77 | 8.161 ( 6.517 - 10.22 ) | 8.097 (469.761) | 3.010 (2.404) | 8.057 (6.674) |
|  | Ejection Fraction Decreased* | 38 | 6.43 ( 4.672 - 8.85 ) | 6.406 (167.369) | 2.674 (1.943) | 6.382 (4.886) |
|  | Aspartate Aminotransferase Increased* | 50 | 2.813 ( 2.13 - 3.715 ) | 2.802 (56.179) | 1.485 (1.124) | 2.799 (2.217) |
|  | Hepatic Enzyme Increased | 56 | 2.434 ( 1.871 - 3.166 ) | 2.425 (45.505) | 1.276 (0.981) | 2.422 (1.944) |
|  | **Blood Alkaline Phosphatase Increased ^§^*** | **33** | **3.918 ( 2.783 - 5.518 )** | **3.907 (68.386)** | **1.963 (1.394)** | **3.899 (2.928)** |
|  | Liver Function Test Abnormal | 27 | 2.267 ( 1.554 - 3.309 ) | 2.263 (17.795) | 1.177 (0.807) | 2.261 (1.648) |
|  | Gamma-Glutamyltransferase Increased ^§^ | 19 | 2.709 ( 1.727 - 4.252 ) | 2.706 (18.738) | 1.434 (0.914) | 2.702 (1.854) |
|  | Blood Potassium Decreased* | 33 | 3.474 ( 2.467 - 4.892 ) | 3.465 (55.347) | 1.790 (1.271) | 3.459 (2.597) |
|  | Transaminases Increased | 20 | 2.519 ( 1.624 - 3.909 ) | 2.516 (16.768) | 1.330 (0.857) | 2.514 (1.741) |
|  | Ejection Fraction Abnormal* | 3 | 6.299 ( 2.026 - 19.584 ) | 6.298 (8.556) | 2.650 (0.852) | 6.275 (2.429) |
|  | Electrocardiogram Change* | 4 | 9.795 ( 3.663 - 26.192 ) | 9.791 (23.233) | 3.283 (1.228) | 9.732 (4.274) |
|  | Neutrophil Count Abnormal | 4 | 4.013 ( 1.504 - 10.71 ) | 4.012 (6.265) | 2.001 (0.750) | 4.003 (1.761) |
|  | Blood Bilirubin Unconjugated Increased* | 3 | 11.558 ( 3.71 - 36.005 ) | 11.554 (19.171) | 3.520 (1.130) | 11.470 (4.432) |
|  | **Blood Chloride Increased ^§^*** | **3** | **6.053 ( 1.947 - 18.816 )** | **6.051 (8.066)** | **2.592 (0.834)** | **6.030 (2.334)** |
|  | Blood Electrolytes Abnormal * | 3 | 7.421 ( 2.386 - 23.082 ) | 7.419 (10.802) | 2.885 (0.928) | 7.386 (2.858) |
| **Blood and Lymphatic System Disorders**  [includes 3 PTs] | Neutropenia* | 194 | 4.023 ( 3.489 - 4.64 ) | 3.955 (426.593) | 1.981 (1.717) | 3.947 (3.503) |
|  | Febrile Neutropenia* | 106 | 5.398 ( 4.455 - 6.54 ) | 5.343 (369.4) | 2.413 (1.992) | 5.327 (4.537) |
|  | **Leukopenia ^§^*** | **50** | **3.047 ( 2.306 - 4.024 )** | **3.035 (66.175)** | **1.599 (1.211)** | **3.030 (2.401)** |
| **Hepatobiliary Disorders**  [includes 9 PTs] | Jaundice* | 64 | 6.495 ( 5.076 - 8.311 ) | 6.454 (288.6) | 2.685 (2.098) | 6.430 (5.232) |
|  | Hepatotoxicity* | 47 | 5.36 ( 4.022 - 7.143 ) | 5.336 (160.86) | 2.411 (1.809) | 5.320 (4.184) |
|  | Hepatic Function Abnormal* | 41 | 3.185 ( 2.342 - 4.33 ) | 3.174 (58.866) | 1.664 (1.224) | 3.170 (2.451) |
|  | Hyperbilirubinemia* | 35 | 10.507 ( 7.529 - 14.661 ) | 10.468 (288.334) | 3.378 (2.421) | 10.399 (7.869) |
|  | Hepatic Failure | 27 | 2.38 ( 1.63 - 3.473 ) | 2.375 (20.141) | 1.247 (0.854) | 2.373 (1.729) |
|  | **Cholangitis ^§^*** | **10** | **5.2 ( 2.794 - 9.679 )** | **5.195 (29.707)** | **2.373 (1.275)** | **5.180 (3.080)** |
|  | **Jaundice Cholestatic ^§^*** | **10** | **8.59 ( 4.612 - 16.001 )** | **8.581 (59.262)** | **3.094 (1.661)** | **8.536 (5.073)** |
|  | Ocular Icterus* | 10 | 6.425 ( 3.451 - 11.962 ) | 6.419 (40.312) | 2.677 (1.438) | 6.395 (3.802) |
|  | **Hepatic Pain ^§^*** | **6** | **4.097 ( 1.838 - 9.133 )** | **4.095 (11.08)** | **2.031 (0.911)** | **4.086 (2.090)** |
| **Infections and Infestations**  [includes 13 PTs] | **Cellulitis ^§^*** | **56** | **3.365 ( 2.586 - 4.377 )** | **3.349 (89.908)** | **1.741 (1.339)** | **3.344 (2.683)** |
|  | Paronychia* | 55 | 43.349 ( 33.123 - 56.731) | 43.076 (2154.634) | 5.387 (4.117) | 41.858 (33.420) |
|  | Nail Infection* | 23 | 52.949 ( 34.908 - 80.316) | 52.81 (1078.411) | 5.672 (3.739) | 50.982 (35.976) |
|  | Rash Pustular* | 19 | 7.66 ( 4.878 - 12.03 ) | 7.646 (102.69) | 2.928 (1.864) | 7.611 (5.217) |
|  | **Gastroenteritis ^§^*** | **20** | **4.441 ( 2.862 - 6.892 )** | **4.433 (49.663)** | **2.145 (1.382)** | **4.422 (3.062)** |
|  | **Erysipelas ^§^*** | **15** | **9.99 ( 6.009 - 16.607 )** | **9.974 (111.549)** | **3.309 (1.991)** | **9.912 (6.479)** |
|  | Wound Infection ^§^ | 7 | 2.612 ( 1.244 - 5.484 ) | 2.61 (5.427) | 1.383 (0.658) | 2.607 (1.402) |
|  | **Clostridial Infection ^§^*** | **8** | **4.952 ( 2.473 - 9.917 )** | **4.948 (21.337)** | **2.303 (1.150)** | **4.935 (2.760)** |
|  | **Enteritis Infectious ^§^*** | **5** | **9.62 ( 3.992 - 23.187 )** | **9.615 (30.257)** | **3.257 (1.351)** | **9.558 (4.578)** |
|  | **Enterocolitis Infectious ^§^*** | **3** | **8.791 ( 2.825 - 27.357 )** | **8.788 (13.564)** | **3.128 (1.005)** | **8.741 (3.381)** |
|  | Escherichia Urinary Tract Infection^§^ | 4 | 3.228 ( 1.21 - 8.613 ) | 3.227 (4.113) | 1.688 (0.633) | 3.222 (1.418) |
|  | **Hepatic Infection ^§^*** | **6** | **13.423 ( 6.006 - 29.997 )** | **13.414 (56.546)** | **3.733 (1.671)** | **13.300 (6.786)** |
|  | **Diarrhea Infectious ^§^*** | **3** | **12.899 ( 4.138 - 40.205 )** | **12.895 (21.895)** | **3.677 (1.180)** | **12.790 (4.940)** |
| **Respiratory, Thoracic and Mediastinal Disorders**  [includes 12 PTs] | Pleural Effusion ^§^ | 47 | 2.325 ( 1.745 - 3.098 ) | 2.318 (33.928) | 1.211 (0.909) | 2.316 (1.821) |
|  | Epistaxis* | 77 | 2.952 ( 2.359 - 3.696 ) | 2.935 (96.396) | 1.551 (1.239) | 2.931 (2.429) |
|  | Lung Infiltration ^§^ | 8 | 2.537 ( 1.268 - 5.079 ) | 2.536 (5.976) | 1.341 (0.670) | 2.533 (1.418) |
|  | Pulmonary Thrombosis ^§^ | 12 | 3.011 ( 1.708 - 5.306 ) | 3.008 (14.114) | 1.587 (0.900) | 3.004 (1.869) |
|  | **Nasal Dryness ^§^*** | **10** | **4.627 ( 2.486 - 8.612 )** | **4.623 (24.81)** | **2.205 (1.185)** | **4.611 (2.742)** |
|  | Rales ^§^ | 6 | 3.421 ( 1.535 - 7.625 ) | 3.42 (7.977) | 1.771 (0.795) | 3.414 (1.746) |
|  | **Hypercapnia ^§^*** | **5** | **5.387 ( 2.238 - 12.968 )** | **5.385 (13.685)** | **2.425 (1.007)** | **5.369 (2.574)** |
|  | **Hypocapnia ^§^*** | **5** | **46.917 (19.249 -114.354)** | **46.89 (175.295)** | **5.506 (2.259)** | **45.447 (21.566)** |
|  | **Pleuritic Pain ^§^*** | **5** | **5.198 ( 2.159 - 12.51 )** | **5.195 (12.955)** | **2.373 (0.986)** | **5.180 (2.484)** |
|  | **Nasal Ulcer ^§^*** | **6** | **11.117 ( 4.977 - 24.828 )** | **11.109 (45.199)** | **3.464 (1.551)** | **11.032 (5.632)** |
|  | **Oropharyngeal Blistering ^§^*** | **4** | **5.376 ( 2.014 - 14.353 )** | **5.374 (10.162)** | **2.422 (0.907)** | **5.358 (2.356)** |
|  | **Nasal Septum Perforation ^§^*** | **3** | **12.106 ( 3.885 - 37.723 )** | **12.102 (20.286)** | **3.586 (1.151)** | **12.010 (4.640)** |
| **Cardiac Disorders**  [includes 6 PTs] | Cardiotoxicity* | 25 | 7.234 ( 4.88 - 10.722 ) | 7.215 (127.118) | 2.845 (1.919) | 7.184 (5.169) |
|  | **Pericardial Effusion ^§^*** | **28** | **3.521 ( 2.429 - 5.105 )** | **3.513 (47.773)** | **1.810 (1.248)** | **3.507 (2.570)** |
|  | Pneumonitis | 22 | 2.527 ( 1.663 - 3.842 ) | 2.523 (18.725) | 1.334 (0.877) | 2.521 (1.776) |
|  | Left Ventricular Dysfunction* | 11 | 5.104 ( 2.822 - 9.229 ) | 5.098 (32.149) | 2.346 (1.297 | 5.084 (3.097) |
|  | **Arteriospasm Coronary ^§^*** | **7** | **4.649 ( 2.213 - 9.766 )** | **4.646 (16.496)** | **2.212 (1.053)** | **4.634 (2.490)** |
|  | Cardiopulmonary Failure* | 6 | 4.125 ( 1.85 - 9.194 ) | 4.122 (11.207) | 2.040 (0.915) | 4.114 (2.103) |
| **Injury, Poisoning and Procedural Complications**  [includes 4 PTs] | **Skin Laceration ^§^*** | **30** | **4.247 ( 2.966 - 6.081 )** | **4.235 (70.791)** | **2.079 (1.452)** | **4.225 (3.129)** |
|  | Wound Secretion ^§^ | 5 | 4.119 ( 1.712 - 9.911 ) | 4.117 (8.862) | 2.038 (0.847) | 4.108 (1.970) |
|  | **Radiation Pneumonitis ^§^*** | **4** | **5.578 ( 2.089 - 14.895 )** | **5.576 (10.751)** | **2.475 (0.927)** | **5.559 (2.444)** |
|  | **Radiation Skin Injury ^§^*** | **4** | **10.546 ( 3.943 - 28.207 )** | **10.542 (25.473)** | **3.389 (1.267)** | **10.473 (4.598)** |
| **Psychiatric Disorders**  [includes 1 PT] | **Eating Disorder ^§^*** | **28** | **3.627 ( 2.501 - 5.258 )** | **3.618 (50.375)** | **1.853 (1.278)** | **3.611 (2.647)** |
| **Nervous System Disorders**  [includes 2 PTs] | Brain Oedema ^§^ | 13 | 2.896 ( 1.68 - 4.993 ) | 2.893 (14.247) | 1.531 (0.888) | 2.890 (1.832) |
|  | **Hyperesthesia ^§^*** | **12** | **3.93 ( 2.23 - 6.929 )** | **3.926 (23.271)** | **1.970 (1.118)** | **3.918 (2.438)** |
| **Vascular Disorders**  [includes 2 PTs] | **Lymphoedema ^§^*** | **13** | **5.912 ( 3.427 - 10.196 )** | **5.904 (47.977)** | **2.557 (1.482)** | **5.884 (3.729)** |
|  | Phlebitis ^§^ | 5 | 2.969 ( 1.234 - 7.142 ) | 2.968 (4.695) | 1.568 (0.652) | 2.964 (1.422) |
| **Renal and Urinary Disorders**  [includes 1 PT] | Oliguria ^§^ | 6 | 2.81 ( 1.261 ; 6.262 ) | 2.809 (5.287) | 1.488 (0.668) | 2.805 (1.435) |
| **Reproductive System and Breast Disorders**  [includes 1 PT] | Breast Discharge ^§^ | 3 | 4.151 ( 1.336 - 12.895 ) | 4.15 (4.355) | 2.050 (0.660) | 4.141 (1.604) |
